# Expanded Hepatic Progenitor Cells Featured with Aggregation of α‐Synuclein Contribute to Pathologic Bile Duct Regeneration in Biliary Atresia

**DOI:** 10.1002/advs.76054

**Published:** 2026-06-29

**Authors:** Hua Xie, Mengyan Zhu, Zhongxian Zhu, Jiaqi Tang, Ruyi Zhang, Zequan Ding, Junzhi Li, Zhongluan Wu, Paul Kwong Hang Tam, Qianghu Wang, Vincent Chi Hang Lui, Yankai Xia, Weibing Tang

**Affiliations:** ^1^ Department of Pediatric Surgery Children's Hospital of Nanjing Medical University Nanjing China; ^2^ Department of Bioinformatics School of Basic Medical Sciences Nanjing Medical University Nanjing China; ^3^ Department of Surgery School of Clinical Medicine The University of Hong Kong Hong Kong SAR China; ^4^ Faculty of Medicine Macau University of Science and Technology Macau SAR China; ^5^ School of Public Health Key Laboratory of Modern Toxicology of Ministry of Education Nanjing Medical University Nanjing China

**Keywords:** bile duct regeneration, biliary atresia, hepatic progenitor cells, reactive ductular cells, α‐synuclein

## Abstract

Ductular reaction (DR) is a hallmark of biliary atresia (BA), but its underlying mechanisms remain unclear. In this study, we identified an expanded NCAM1^+^EpCAM^+^ hepatic progenitor cells (HPCs) as the predominant DR‐related cell cluster using single‐nucleus RNA sequencing, and NCAM1^+^EpCAM^+^ HPC‐derived organoids exhibited impaired biliary differentiation in BA. α‐synuclein was characterized as the signature gene of this cluster, exhibiting upregulated expression in liver tissues from both human BA and mouse BA models, as well as in the serum of patients with BA. Biliary organoids derived from mouse single cells and human induced pluripotent stem cells (iPSCs) confirmed that α‐synuclein accumulation drove aberrant biliary development in both mouse and human organoids, and induced BA‐like transcriptomic alterations, characterized by significant enrichment of the glutathione (GSH) metabolic pathway. Consistently, hepatic GSH was reduced in both human BA and mouse BA models. In human intrahepatic biliary epithelial cells (HiBECs), α‐synuclein overexpression decreased cellular GSH content and increased mitochondrial reactive oxygen species (ROS) under oxidative stress. The present study showed that NCAM1^+^EpCAM^+^ HPCs were expanded and characterized by an aggregation of α‐synuclein in BA, which could increase cellular susceptibility to GSH‐associated redox imbalance, and lead to aberrant bile duct regeneration.

## Graphical abstract

Expanded NCAM1^+^EpCAM^+^ hepatic progenitor cells in biliary atresia are characterized by aggregation of α‐synuclein. This pathological protein potentiates cellular susceptibility to GSH‐dependent redox dyshomeostasis, induces unstable biliary cell fate specification, and subsequently drives aberrant biliary regeneration.

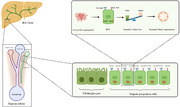

## 1 Introduction

Biliary atresia (BA) is a life‐threatening cholangiopathy in infants and is characterized by obstructive intra‐ and extra‐hepatic bile ducts and progressive liver fibrosis [1]. The Kasai portoenterostomy (KPE) is currently the primary treatment for BA. The KPE involves resection of obliterated extrahepatic bile ducts and reconstruction of bile drainage with an intestinal conduit. However, because intrahepatic cholangiopathy remains unresolved, < 50% of patients with BA achieve long‐term native liver survival [2]. Therefore, the pathophysiologic characteristics of intrahepatic bile ducts in BA need to be elucidated.

The intrahepatic pathologic changes in BA are considered to be an exaggerated response to injury. Following injury to the biliary tree, cells with a biliary phenotype expand in the portal area of the liver and organize into irregularly shaped structures; the process is known as the ductular reaction (DR) [3]. During the DR process, molecular and cellular pathways that recapitulate embryonic biliary development are reactivated to achieve biliary repair and regeneration. However, due to prolonged injury, the DR ultimately leads to pathologic biliary repair and regeneration [4]. In cholangiopathies including BA, expanded DR in the portal area is one of the most significant pathologic features [5]. Moreover, the DR has been described as the ‘pacemaker’ of biliary fibrosis due to its aberrant crosstalk with fibroblasts by interacting with the extracellular matrix (ECM) and contributing to collagen deposition [6]. Hence, clarifying the features of the DR in BA will afford significant insights into the pathophysiologic mechanisms underlying disease progression.

The DR is described as massive cholangiocyte proliferation (marked by KRT7 or KRT19) or hepatic progenitor cell (HPC) proliferation (marked by EpCAM), depending on the pathology [3]. After loss of biliary homeostasis, massive bile duct hyperplasia is characterized as a DR in cholangiopathies, while HPCs comprise the main component of the DR under severe bile duct injury [7]. HPC activation in the liver is beneficial in promoting liver regeneration. However, it can also trigger liver fibrosis and the formation of tumors. The numbers of SOX9^+^ and PROM1^+^ progenitor cells in BA, which possesses regenerative potential in vivo, are significantly correlated with liver fibrosis, suggesting that the accumulation of dysfunctional HPCs in the liver could participate in BA progression [8, 9]. However, the characteristic of DR‐associated HPCs in the livers of patients with BA has not been fully elucidated.

In the current study, a population of EpCAM^+^ HPCs with high NCAM1 expression was shown to be enriched in BA liver tissues through single‐nucleus RNA sequencing. Organoids derived from NCAM1^+^EpCAM^+^ HPCs showed impaired growth and disrupted biliary lineage determination. Furthermore, Parkinson disease‐associated protein α‐synuclein, which serves as the featured gene of NCAM1^+^EpCAM^+^ HPCs, was shown to be aggregated surrounding the intrahepatic HPCs in BA livers. Moreover, the total serum α‐synuclein may serve as a potential biomarker for BA. Finally, the iPSC‐derived cholangiocyte‐like cell (CLC) organoid model showed that CLC organoids with accumulation of α‐synuclein had unstable biliary differentiation and partially resembled the morphologic and transcriptomic characteristics of CLC organoids derived from BA liver tissue. The current study revealed the following: (i) new characteristic features of HPCs in BA liver tissue; and (ii) new molecule targets to reduce the accumulation of abnormal HPCs to improve biliary regeneration in BA.

## 2 Methods

### 2.1 Human Liver and Serum Samples

In this study, liver and serum samples were obtained from patients with type III BA and disease controls including choledochal cyst (CC) or neonatal intrahepatic cholestasis (IHC) treated in Children's Hospital of Nanjing Medical University from January 2019 to September 2025. The diagnosis of BA was based on operative cholangiography and followed anatomy of hepatic hilar region. Liver samples of BA were obtained at the time of KPE, while control samples were obtained during hepaticojejunostomy for CC or diagnostic cholangiography for IHC. Once obtaining the samples, liver tissues were rinsed with ice‐cold Dulbecco's phosphate‐buffered saline (D‐PBS) and divided into three portions. One portion was fixed in paraformaldehyde for subsequent preparation of paraffin‐embedded liver tissue sections. The second portion was preserved in tissue storage solution (Miltenyi Biotec, USA) for magnetic bead sorting and organoid culture. The remaining portion was snap‐frozen and stored at −80°C for further analyses. Serum samples from each subject were obtained at the time of admission. This study obtained approval from the Ethics Committee of the Children's Hospital of Nanjing Medical University (approval number: 202103031), and conducted according to the ethical guidelines of the Declaration of Helsinki.

### 2.2 Single‐Nucleus RNA Sequencing (snRNA‐seq)

Single‐nucleus samples were prepared from frozen liver tissues from 4 patients with BA and 4 patients with choledochal cyst during December 2019 and March 2020. No matching was done between BA and controls. Clinical parameters including sex assigned at birth, age at surgery, alanine transaminase (ALT), aspartate transaminase (AST), gamma‐glutamyl transferase (GGT), and direct bilirubin (DBIL) were listed in Table S1. Single nucleus extraction and data processing are detailed in the Supporting Methods.

### 2.3 NCAM1^+^ EpCAM^+^ and NCAM1^−^ EpCAM^+^ Cells Sorting

We previously have generated EpCAM^+^ cells derived liver organoids from BA and controls [10]. In this study, fresh liver biopsy samples from 3 patients with BA (clinical information was summarized in Table S2) were included in the step‐wise magnetic cell sorting and organoids culture. First, liver samples were digested using a single cell dissociator (DSC‐410; RWD, China). After single cells (viability>80%) were obtained, NCAM1 (CD56)‐positive and ‐negative expressing cells were isolated according to the REAlease CD56 MicroBead kit manual (130‐117‐033; Miltenyi, USA). Briefly, cells were incubated in 10 µL of REAlease CD56‐biotin reagent for 5 min at 25°C to label NCAM1‐expressing cells after removing digestive enzymes and red blood cells (RBCs). Then, biotin‐labeled cells were incubated in 50 µL of REAlease Anti‐biotin MicroBeads for 5 min at 25°C. The cells were then sorted using a magnetic column. NCAM1 unlabeled and labeled cells in the column were flushed with 5 mL of separation buffer (130‐091‐376 diluted with MACS BSA stock solution; Miltenyi) with autoMACS Rinsing Solution (130‐091‐222; Miltenyi) at a 1:20 ratio. NCAM1 unlabeled and labeled cells were resuspended in 5 mL of separation buffer; 100 µL of REAlease Release Reagent was added to release biotin. The cells were resuspended in 5 mL cold Running buffer (same as separation buffer) and magnetic labelling was performed using CD326 (EpCAM) MicroBeads (130‐061‐101; Miltenyi). NCAM1 unlabeled and labeled cells were incubated with CD326 (EpCAM) MicroBeads at 4°C for 30 min. NCAM1 unlabeled and labeled EpCAM^+^ cells in the column were flushed with 5 mL cold Running buffer and marked as NCAM1^+^ EpCAM^+^ or NCAM1^−^ EpCAM^+^ cells. The cells of each group were seeded in 50 µL Matrigel (356231, Corning) with 2 wells at least and cultured in organoid medium.

### 2.4 Immunofluorescence and Immunohistochemical Analysis

The Opal 4‐Color Manual IHC Kit (PerkinElmer) was used for multiple immunofluorescences, according to the manufacturer's instructions. In brief, human liver or organoid sections were first deparaffinized and rehydrated, followed by primary antibody staining one day by one or by only 1% Thioflavin‐S (T1892, Sigma). Antigen retrieval was conducted by heating slides in 10 mM sodium citrate buffer (pH 6.0) at 100°C for 5 min. Prior to primary antibody incubation, all sections were blocked with a blocking buffer (PBS containing 10% normal donkey serum) for 1 h at room temperature. Sections were then incubated with appropriate fluorescent secondary antibodies in blocking buffer for 45 min. After DAPI staining, sections were mounted using anti‐fade mounting medium, and images were captured with a ZEISS Axiovert 5 microscope (Germany). For immunohistochemical analysis, paraffin sections were dewaxed, subjected to antigen retrieval, and blocked with 3% BSA, followed by incubation with NCAM1 antibody at 4°C overnight in a humidified chamber. After three washes with PBS, sections were incubated with HRP‐conjugated secondary antibody (YFSA02, Yfxbio Biotech, China) for 1 h at 25°C. PBS‐washed sections were developed with DAB substrate (ADK001, Abcepta Biotech, China). Images were captured using Pannoramic MIDI digital scanner. Quantification of NCAM1 expression was performed using the AIpathwell IHC Analysis Software (Servicebio, China). Briefly, images of at least 3 portal areas per section were captured under 10× magnification. H‐scores were calculated for each image, and the average value was used as the final H‐score. Detailed information including Research Resource Identifier (RRID) on primary and secondary antibodies is provided in Table S3.

### 2.5 Serum Total α‐synuclein Detection by Enzyme‐Linked Immunosorbent Assay

Total serum α‐synuclein was detected using an enzyme‐linked immunosorbent assay (ELISA) kit from Cusabio (CSB‐E18033h; China). Serum samples from 40 BA and 20 controls were included and the detailed clinical information of samples from the two groups is included in Table S4. The frozen serum samples were thawed on ice. A series of gradient dilutions of the standards were prepared according to the kit instructions. The standards and serum samples to be tested were added to 96‐well plates. Biotinylated antibody working, horseradish peroxidase (HRP)‐conjugated streptavidin working, substrate, and stop solutions were sequentially added to each well. Following reaction termination, the OD value of each well was measured at 450 nm with a microplate reader within 5 min. A standard curve was generated based on the OD values of the standards, and the serum protein concentrations in each sample were calculated according to the standard curve.

### 2.6 Preparation and Usage of α‐synuclein Preformed Fibrils (PFFs)

Human PFFs (SPR‐322, 500 µg; Canada) and mouse PFFs (SPR‐324, 100 µg; Canada) were purchased from StressMarq with a stock concentration of 5 mg/mL (357 µm) and stored at −80°C. The first step involved preparing a 100 µm working solution. The stock solution was retrieved from the −80°C freezer and dissolved at room temperature. To prepare the 100 µm solution, 100 µL of the stock solution was mixed with 257 µL of D‐PBS. The mixture was then sonicated in a water bath at room temperature for 5 min. The solution was aliquoted into 50 µL portions and stored at −80°C. A 50 µL aliquot was thawed at room temperature. In a 1.5 mL EP tube, the 50 µL aliquot is diluted with 450 µL of D‐PBS to achieve a final concentration of 10 µm. The solution was then sonicated at 30% amplitude (1 s on and 1 s off) for 60 cycles (total time = 2 min). After sonication, the solution was filtered through a 0.22 µm filter. The filtered solution was subsequently diluted into the culture medium to achieve a final concentration of 100 nm.

### 2.7 iPSC Differentiation

The iPSC cell line was generated from ethnic Chinese healthy male peripheral blood mononuclear cells (PBMCs). Whole‐genome sequencing of the PBMCs from which the iPSCs were derived and the sequencing results confirmed there were no chromosomal deletions, insertions, or rearrangements being introduced during iPSC derivation. The differentiation of iPSC to cholangiocyte progenitors (CPs) and CLC organoids followed a published step‐wise differentiation protocol with minor modifications; specifically, the organoid culture medium was replaced with medium same as liver tissue‐derived organoids [11]. Detailed reagents for iPSC maintenance are provided in Table S5. PFFs at a final concentration of 100 nm were added to the differentiation medium and the medium was changed every 24 h from Hepatoblasts (HBs) stage to CPs stage. After being digested into single cells, the CPs were divided into three parts, which were respectively used for single cell RNA sequencing, Western blot analysis, and the further culture of CLC organoids on day 16. Every 20 000 CPs were seeded in 50 µL Matrigel (356237, Corning) with 8 wells at least.

### 2.8 CLC Organoids Culture and Harvest

Mouse single cell derived CLC organoids culture was conducted as we reported previously [12]. Briefly, organoids at the third passage (P3) were dissociated with 1 mL of Cell Recovery Solution (354253, Corning) for Matrigel removal. The dissociated organoid fragments were collected into 15 mL centrifuge tubes following centrifugation at 444 × g for 5 min. Subsequently, 1 mL of pre‐warmed TrypLE Express (12604013, Gibco) was added to the pellet, and the suspension was incubated at 37°C for 5 min. Following incubation, the enzymatic reaction was quenched by adding 1 mL of fetal bovine serum (30044333, Gibco). The mixture was then triturated 40–50 times using a glass capillary (0.3–0.5 mm in diameter) to dissociate organoids into a single‐cell suspension. The magnetic sorted cells, the CPs, or the dissociated mouse single cells were washed 2–3 times with ice cold advanced DMEM/F12 medium containing 1% P/S, and the supernatant was discarded. Matrigel (50 µL/up to 2 × 10^4^ cells) was used to resuspend the pellet with a cold pipette tip avoiding air bubbles. Fifty microliters of the cell/Matrigel mixture were pipetted into each well. The plates were kept in a 37°C cell culture incubator for 5 min. The plate was flipped and kept in an incubator for 15 min to allow the Matrigel to solidify completely. Organoid medium (Table S6) was pre‐warmed in a 37°C water bath for 5 min. For PFFs treated groups, CLC organoids culture medium was supplemented with human or mouse PFFs (100 nm) from day 17 to 25 (iPSC‐derived CLC organoids) or day 0 to 7 (mouse single cell‐ derived CLC organoids). The ROCK inhibitor, Y27632, was added to each well for the first 6 days of culture. The medium was changed every 2 days.

For harvesting the CLC organoids for Smart‐seq: After discarding the culture medium from the 24‐well or 4‐well plate, 1 mL of ice‐cold PBS was added to harvest the organoids. The Matrigel was gently scraped and the suspension was slowly aspirated and pipetted repeatedly to avoid disrupting the morphology of the organoids. The solution containing the organoids was transferred into a Petri dish with 1 mL of PBS and incubated on ice for 15 min to fully dissolve the Matrigel for organoid selection and Smart‐seq. The organoids were aspirated with a 10‐µL pipette tip under a microscope and subsequently transferred into an 8‐tube rack containing lysis buffer.

For harvesting the CLC organoids for immunofluorescent staining, the Matrigel was gently scraped by 1 mL ice‐cold PBS and collected in a 1.5‐mL EP tube. EP tubes were inserted into ice for 15 min after adding 10 mL more ice‐cold PBS to completely remove the Matrigel. In cases in which a larger number of organoids were present, organoids sedimented to the bottom of the tube. If the organoid quantity was low, the solution was centrifuged at 200 g for 10 s and the supernatant was discarded to avoid loss. The organoids were then fixed with 500 µL of 4% PFA for 20–30 min on ice. The supernatant containing PFA was discarded as described above and 200 µL of Histogel (HG4000012; Epredia, USA) was added to the tube. The Histogel with the organoids was dehydrated through a graded alcohol series after incubating at ‐20°C for 10 min and embedded in paraffin for staining. For whole‐mount staining of organoids, the blocking buffer (5% BSA + 0.1% Triton X‐100) was added immediately after PFA removal, followed by overnight incubation. On the next day, the blocking buffer was discarded, and primary antibodies were added for a 24‐h incubation. On the third day, secondary antibodies were applied and incubated for 2 h. Finally, organoids were resuspended in DAPI solution, dropped into a confocal dish, and left to stand for 24 h in the dark, to allow organoids to settle to the bottom of the dish for confocal imaging.

### 2.9 Single‐Cell RNA Sequencing (scRNA‐seq) for Cholangiocyte Progenitors

Single‐cell suspensions were prepared from PFF‐treated and ‐untreated CPs using 10x Genomics Single‐cell 3′ v2 and 3′ v3 Reagent kits following the manufacturer's protocol. Cells were stained with 0.4% trypan blue (ThermoFisher Scientific, USA) to check the viability on a Countess II Automated Cell Counter (ThermoFisher Scientific). A full description of the 10x sample and data processing is included in the Extended Methods.

### 2.10 Smart‐seq

CLC organoids derived from distinct experimental groups were isolated from Matrigel and transferred to individual microtubes for RNA sequencing. Organoid lysis, total RNA extraction, reverse transcription, amplification, and library preparation were conducted using a single‐cell RNA‐sequencing platform (Smart‐seq2.0) with subtle adjustments to produce bulk RNA transcriptomes and attain adequate depth in transcriptome profiling. A description of data processing is detailed in the Supporting Methods.

### 2.11 Statistics

Statistical analysis and visualization of the data were performed using R software (version 3.6.3) and Prism 9.4.1 (GraphPad, La Jolla, CA), with detailed statistical information provided in the respective figure legends or methods section. Continuous variables are presented as mean ± standard deviation (SD). A chi‐square test or Fisher's exact test was applied to categorical data, and Student's *t*‐test was used for comparing two groups. Two‐way analysis of variance (ANOVA) was used to assess the effects of two independent variables across multiple groups. The diagnostic efficacy of the biomarkers was evaluated utilizing receiver operating characteristic (ROC) curve analysis and the corresponding area under the curve (AUC) values. The optimal cut‐off threshold, determined via the Youden index method, was employed. Statistical significance was defined as *p* < 0.05.

## 3 Results

### 3.1 snRNA‐seq of Human Livers Revealed Expansion of Unique Biliary Clusters in BA

**FIGURE 1:**
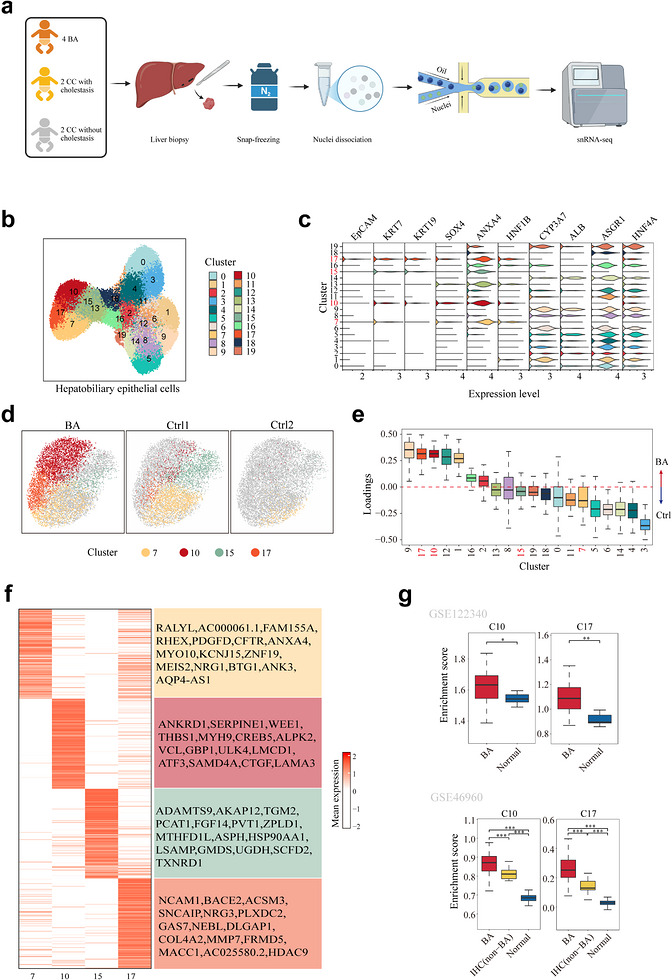
Expanded biliary clusters in BA as revealed by single‐nucleus RNA sequencing. (a) Workflow of the single‐nucleus RNA sequencing of the liver biopsies from four patients with BA and four choledochal cysts controls. Four controls were divided into two subgroups: CC with severe cholestasis (2 cases) and CC without severe cholestasis (2 cases). (b) Uniform manifold approximation and projection (UMAP) visualization of cells from integrated BA and control liver samples colored according to cluster. (c) Violin plot of hepatic and biliary marker genes expression of clusters. (d) UMAP visualization of biliary cells of BA, Ctrl1 and Ctrl2. (e) Case‐Control Analysis (CACOA) for BA enriched clusters, with biliary clusters highlighted in red. (f) Heatmap of DEGs (log_2_FC>0.25, adjusted *p* <0.05) of the biliary clusters. (g): Gene Set Variation Analysis of DEGs of cluster 10 and cluster 17 in the extended dataset (GSE46960 included 64 cases of BA, 14 cases of non‐BA intrahepatic cholestatic liver tissues, and 7 cases of normal liver tissues. GSE122340 contained 171 BA and 7 normal liver tissues. *P* value was calculated by wilcoxon rank‐sum test. *p < 0.05; **p < 0.01; ***p < 0.001. Abbreviation: Ctrl1, choledochal cysts with severe cholestasis; Ctrl2, choledochal cysts without severe cholestasis.

To elucidate the characteristics of the DR in BA, snRNA‐seq was used to generate transcriptomes of individual cells in liver biopsies from four patients with BA and four patients with choledochal cyst (CC) (Figure 1a). Based on the presence or absence of severe cholestasis in liver tissues, the CC samples were divided into two subgroups: two patients with severe cholestasis (Ctrl1), and two patients without severe cholestasis (Ctrl2) (Table S1). After quality control and batch effect correction (Figure S1a,b), a total of 106,149 cells were annotated into 6 major cell types, including hepatobiliary epithelial cells (marked by *HNF1B*, *ALB*, *HNF4A*, and *ANXA4*), fibroblast/stellate cells (marked by *ACTA2*, *COL1A1*, *DCN*, and *SPARCL1*), endothelial cells (marked by *PECAM1*, *CD36*, *ENG*, and *KDR*), macrophages (marked by *CD68*, *MAFB*, *CD86*, and *CD163*), T and natural killer (NK) cells (marked by *CD3D*, *CD96*, *KLRD1*, and *CD247*), and B cells (marked by *EBF1*, *MS4A1*, *IGHM*, and *CD79A*; Figure S1c,d).

Re‐clustering of the hepatobiliary epithelial cells identified 20 clusters (cluster 0–19; Figure 1b). Cluster 7, 10, 15, and 17 were identified as biliary clusters based on the high level of biliary marker (*KRT19*, *KRT7*, and *HNF1B*) expression; cluster 17 also had high *EpCAM* expression. Other clusters were defined as hepatic populations based on the expression of hepatocyte markers (*HNF4A*, *ALB*, *CYP3A7*, and *ASGR1*; Figure 1c). Comparative analysis of Ctrl1, Ctrl2, and BA biliary clusters showed that cluster 10 and 17 were highly enriched in BA. Cluster 15 was slightly enriched in Ctrl1, while cluster 7 was similarly present in all groups (Figure 1d). Case‐Control Analysis (CACOA) further confirmed that clusters 10 and 17 were enriched in BA (Figure 1e). The heatmap showed transcriptomic differences between the biliary clusters (Figure 1f). Differentially expressed genes (DEGs) with a log_2_fold change (FC) > 0.25 and adjusted *p* < 0.05 of clusters 10 and 17 were also found to be significantly enriched in the BA group through validation in two published BA associated datasets (GSE122340 and GSE46960; Figure 1g and Figure S1e,f). The above results revealed that two biliary clusters were distinctly expanded in BA livers.

### 3.2 Cells of the Expanded Biliary Clusters in BA Livers Displayed Features of Ductular Reaction

**FIGURE 2:**
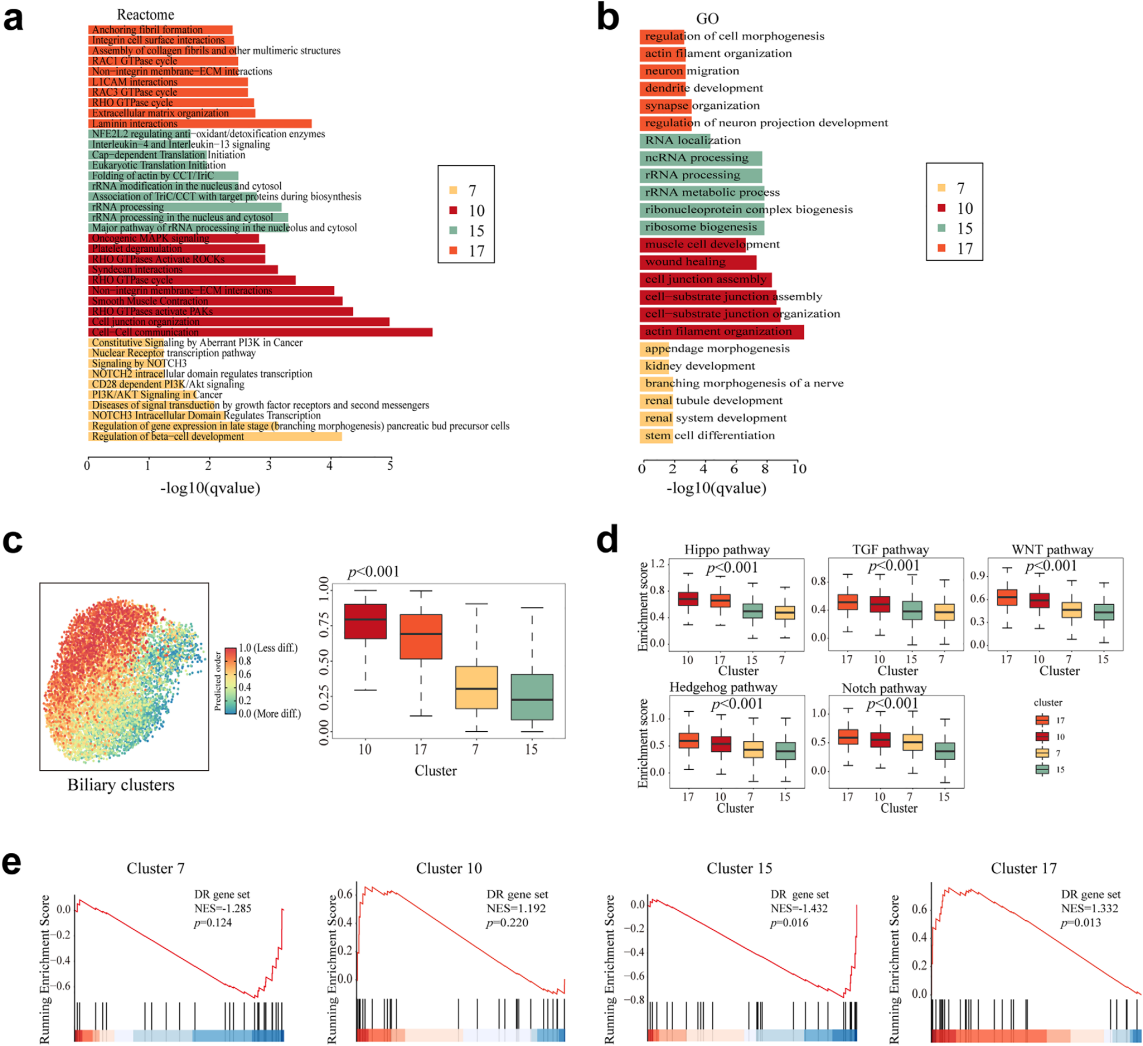
Expanded biliary clusters in BA livers displayed features of ductular reaction. (a) Reactome pathway analysis and (b) Gene Ontology analysis on DEGs of biliary clusters (cluster 7, 10, 15, and 17) from snRNA‐seq data. The color indicated cluster assignment and the bar indicated log_10_(qvalue) calculated by hypergeometric test of each term. q values represent FDR‐adjusted p‐values. (c) Predicted score of each biliary cluster (cluster 7, 10, 15, and 17) analyzed by CytoTRACE. Comparisons among multiple clusters were performed using the Kruskal‐Wallis test, with a significance level of *p* < 0.001. (d) Gene Set Variation Analysis of pathways (Hippo, TGF, WNT, Hedgehog, and Notch) in each biliary cluster. Comparisons among multiple clusters were performed using the Kruskal‐Wallis test, with a significance level of *p* < 0.001. (e) Gene Set Enrichment Analysis of DR related gene set in each biliary cluster. *P* value was calculated by Permutation test. NES: normalized enrichment score. *p* < 0.05 was considered as statistically significant.

To further characterize the two expanded biliary clusters, Gene Ontology (GO) and pathway enrichment analysis were performed on the upregulated DEGs of each biliary cluster. In contrast to cluster 15 and 7, pathway enrichment analysis of DEGs in cluster 10 and 17 revealed significant enrichment in pathways related to repair and regeneration, such as ‘Cell junction’, ‘Wound healing’, and ‘Extracellular matrix organization’. Interestingly, GO enrichment analysis of cluster 17 highlighted the involvement of neuro‐related biological processes (‘regulation of neuron projection development’ and ‘synapse organization’) (Figure 2a,b). CytoTRACE analysis showed that cluster 10 and cluster 17 exhibited higher scores compared to the other 2 clusters, suggesting they exhibited higher differentiation potential (Figure 2c). Enrichment of pathways related to DR (Hippo, TGF, WNT, Hedgehog, and Notch) were also higher in cluster 10 and 17 compared to the other 2 biliary clusters (Figure 2d). Gene Set Enrichment Analysis (GSEA) demonstrated that the gene set representing DR features across different diseases (Supporting Information 1) was only positively and significantly enriched in cluster 17 (*p* = 0.013, Normalized Enrichment Score (NES) = 1.332; Figure 2e).

**FIGURE 3:**
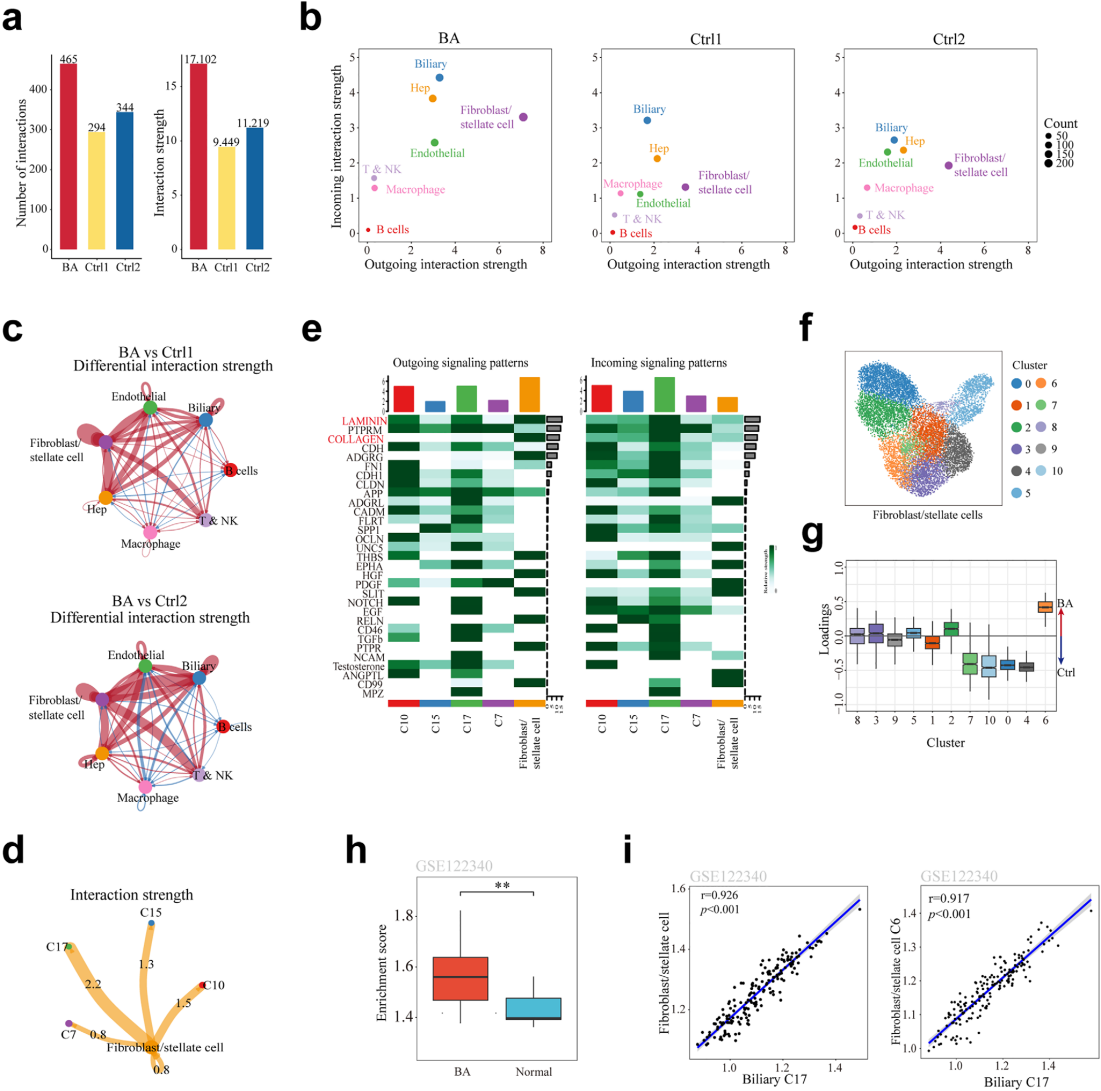
Cell‐cell interaction between biliary clusters and fibroblast/stellate cells. (a) Total interaction strength and number of interaction pairs in BA, Ctrl1 and Ctrl2 samples. (b) Outgoing and incoming interaction strength of each cell type in BA, Ctrl1 and Ctrl2 samples. (c) Interaction strength between each cell type in BA compared with Ctrl1 or Ctrl2 samples. Red lines represented increased interaction strength in BA, blue lines represented decreased interaction strength in BA, and the thickness of the lines showed scores of interaction strength. (d) Interaction strength between each biliary cluster and fibroblast/stellate cells. The thickness of the lines represented the strength of interactions with the scores of interaction strength indicated. (e) Outgoing and incoming interaction signal patterns in each biliary cluster and fibroblast/stellate cells. The bars on the upper side and right side represented the sum of the intensities of the rows and columns. (f) UMAP visualization of fibroblast/stellate cells from integrated BA and control samples colored according to cluster. (g) BA enriched fibroblast/stellate cells cluster revealed by Case‐Control Analysis (CACOA). (h) Gene Set Variation Analysis of DEGs of cluster 6 in the extended dataset (GSE122340 contained 171 BA and 7 normal liver tissues). *P* value was calculated by wilcoxon rank‐sum test. (i) Spearman correlation between values of DEGs in biliary cluster 17 and values of DEGs in fibroblast/stellate cells or fibroblast/stellate cells cluster 6. Each value was assessed by Gene Set Variation Analysis in GSE122340. *p < 0.05; **p < 0.01; ***p < 0.001. Abbreviation: Hep, hepatocyte; Ctrl1, choledochal cysts with severe cholestasis; Ctrl2, choledochal cysts without severe cholestasis.

Ductular cells in DR process can interact with periportal immune, endothelial, and mesenchymal cells in biliary repair and regeneration. Therefore, whether the two BA‐enriched biliary clusters were capable of communication with immune, endothelial, and mesenchymal cells was determined. The number and intensity of cell‐cell interactions were higher in patients with BA (Figure 3a). The biliary clusters had the highest interaction intensity, while the fibroblast/stellate cells exhibited the greatest output interaction intensity, both in BA and controls (Figure 3b). Cell chart showed that BA's biliary clusters exhibited stronger cell‐cell interactions with fibroblast/stellate cells compared to Ctrl1 or Ctrl2 samples, while biliary cluster 17 had the highest interaction intensity (Figure 3c,d). Fibroblast/stellate cells were the primary source of outgoing signaling patterns, whereas biliary cluster 17 was more responsive to incoming signals than other biliary clusters (Figure 3e). Ligands derived from fibroblast/hepatic stellate cells and biliary cluster 17 were predominantly members of the collagen families (COL1A1, COL1A2, COL4A1, COL4A2) and laminin families (LAMC2, LAMA5), which have been confirmed as crucial extracellular matrix components in HBs differentiation [13], exhibited top‐ranked in terms of signal intensity (Figure 3e and Figure S2a,b). To further identify whether biliary cluster 17 interacted with BA's fibroblast/stellate cells, fibroblast/stellate cells were re‐clustered and cluster 6 of fibroblast/stellate cells was significantly enriched in the BA group (Figure 3f,g). The signature of cluster 6 of fibroblast/stellate cells (DEGs with a log_2_FC>0.25 and adjusted *p* < 0.05) was also enriched in the BA‐associated dataset, GSE122340 (Figure 3h). Although few genes overlapped, the signature of fibroblast/stellate cells (DEGs with a log_2_FC>0.25 and adjusted *p* < 0.05) and cluster 6 of fibroblast/stellate cells both exhibited a significantly positive correlation with the signature of biliary cluster 17 (DEGs with a log_2_FC>0.25 and adjusted *p* < 0.05) in the GSE122340 dataset, suggesting expansion of biliary cluster 17 was accompanied by an increase of fibroblast/stellate cells in BA (Figure 3i and Figure S2c,d). Taken together, the cells in biliary cluster 17 presented as the most prominent abnormal DR‐associated biliary cells in BA.

### 3.3 Characterization of NCAM1^+^EpCAM^+^ HPCs in the Expanded Biliary Clusters in BA

**FIGURE 4:**
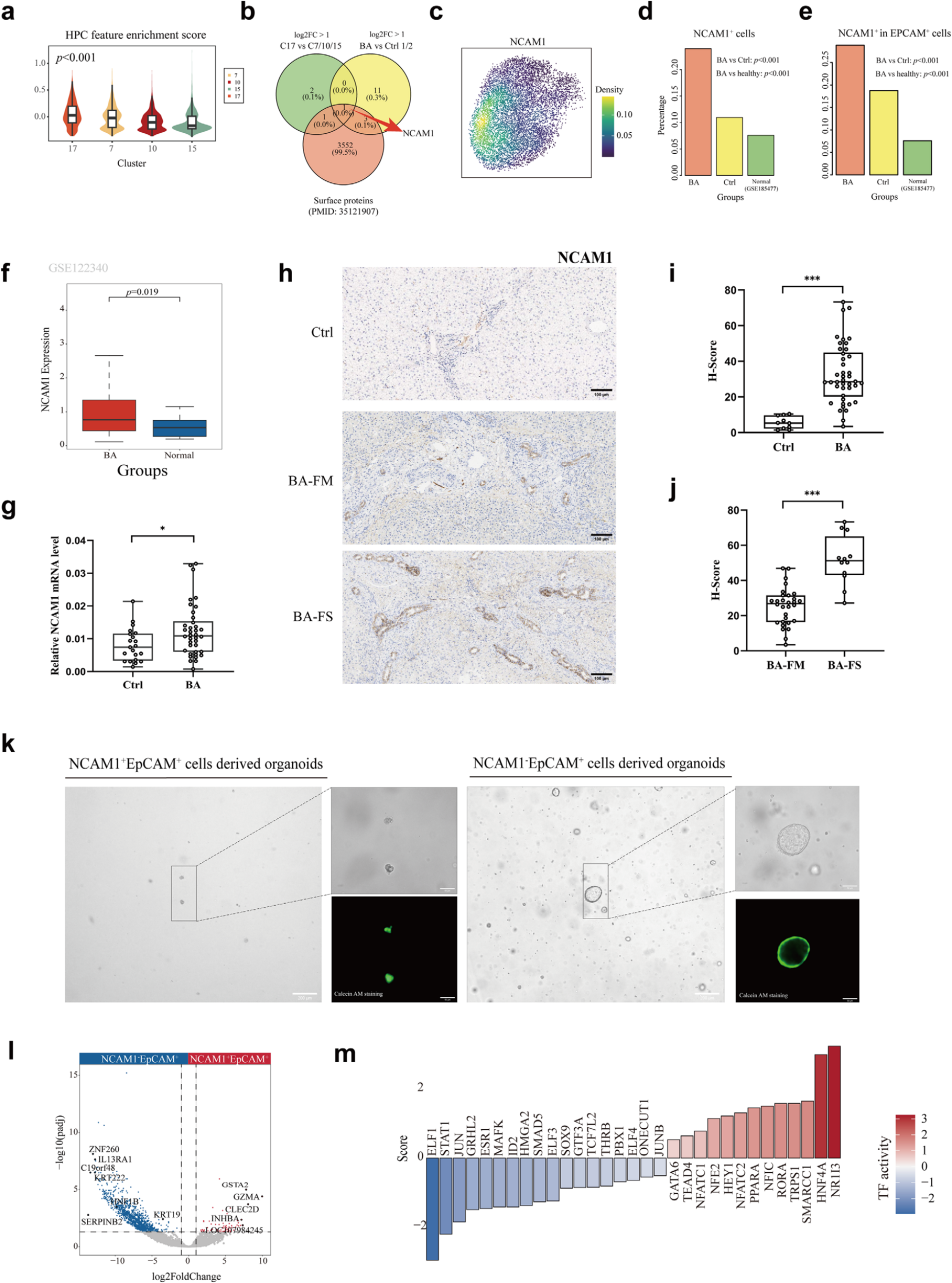
Characterization of NCAM1^+^EpCAM^+^ HPCs in the expanded biliary clusters in BA. (a) Enrichment score of HPCs gene set in each biliary cluster. Comparisons among multiple clusters were performed using the Kruskal‐Wallis test, with a significance level of *p* < 0.001. (b) Venn diagram of DEGs showing higher expression in cluster 17 than other three biliary clusters (avg_log_2_FC>1), the DEGs showing higher expression in BA than the control group across all four biliary clusters (avg_log_2_FC>1), and the membrane protein database. (c) Gene density plot of NCAM1 in biliary clusters. (d): The proportion of NCAM1 positive cells of BA, Ctrl and external single‐cell dataset. *P* value was calculated by Chi‐squared test. (e): The proportion of NCAM1^+^ cells in EpCAM^+^ cells of BA, Ctrl and normal cholangiocyte dataset (GSE185477 contains four healthy human liver caudate lobes). *P* value was calculated by Chi‐squared test. (f): The expression of NCAM1 in BA compared with normal liver tissues in GSE122340 (171 BA and 7 normal liver tissues) calculated by wilcoxon rank‐sum test. (g): The expression of NCAM1 in BA (n = 38) and Ctrl liver tissues (*n* = 21). *P* value was calculated by unpaired t test. (h) Immunohistochemical staining of NCAM1 on paraffin sections of livers from Ctrl, BA with mild liver fibrosis (BA‐FM) and BA with severe liver fibrosis (BA‐FS). Scale bar: 100 µm. (i) Comparison of H‐scores for NCAM1 in portal areas of liver tissues between BA (*n* = 42) and Ctrl (n = 10). *P* value was calculated by unpaired t test. (j) Comparison of H‐scores for NCAM1 in portal areas of liver tissues between BA‐FM (*n* = 30) and BA‐FS (*n* = 12). *P* value was calculated by unpaired t test. (k) Morphology of NCAM1^+^EpCAM^+^ cells derived organoids and NCAM1^−^EpCAM^+^ cells derived organoids in BA (The left side of each group, scale bar: 200 µm). Regions highlighted with dotted boxes were magnified and shown on the right (scale bar: 50 µm). Calcein AM staining (Green) of the organoids were shown. (l) Volcano plot of DEGs of organoids derived from NCAM1^+^EpCAM^+^ cells (*n* = 3) and NCAM1^−^EpCAM^+^ cells (*n* = 4). (m) Bar chart showing transcriptional activity of TFs implicated in biliary and hepatic lineage. Red color indicated increased activity, while blue color indicated decreased activity in NCAM1^+^EpCAM^+^ cells derived organoids.

EpCAM^+^ cells in DR are considered to be HPCs and participate in biliary regeneration [14]. Considering EpCAM expression was significantly higher in cluster 17 among all biliary clusters, we further integrated a gene set of HPCs (*NCAM1*, *LGR5*, *TACSTD2*, *CD24*, *PROM1*, *EpCAM*, *CXCR4*, and *SOX9*). The enrichment score of the gene set was highest in cluster 17, suggesting cluster 17 had the characteristic of HPCs (Figure 4a). However, normal cholangiocytes also express EpCAM, making EpCAM markers alone unable to specifically identify HPCs [15]. To further characterize cluster 17, we intersected the upregulated DEGs in cluster 17 compared with the other three biliary clusters (log_2_FC>1), the upregulated DEGs in BA than the control group across all four biliary clusters (log_2_FC>1), and cell‐surface proteins. *NCAM1* was the only gene that has been screened out (Figure 4b). Therefore, cluster 17 was defined as NCAM1^+^EpCAM^+^ HPCs. The gene density plot showed that *NCAM1* was highly expressed in cluster 17 (Figure 4c). Compared to Ctrl and a published normal cholangiocyte dataset (GSE185477), a significant increase in the proportion of NCAM1^+^ cells was noted in BA, as well as a higher percentage of NCAM1^+^EpCAM^+^ cells in all EpCAM^+^ cells (Figure 4d,e). Moreover, *NCAM1* expression was significantly higher in BA in both GSE122340 and our patient cohort (Figure 4f,g). Immunohistochemical staining showed that NCAM1 expression was significantly increased in the portal area of BA livers, compared with Ctrl livers (Figure 4h,i). Meanwhile, NCAM1 expression was also significantly elevated in BA patients with severe liver fibrosis (BA‐FS), compared with BA patients with mild liver fibrosis (BA‐FM), which was consistent with the finding from single‐cell analysis that expansion of cluster 17 was accompanied by an increase in fibroblast/stellate cells in BA (Figure 4j).

To examine the biliary generation potential of the NCAM1^+^ and NCAM1^−^ EpCAM^+^ cells, a step‐wise dual magnetic beads‐based sorting of NCAM1^+^EpCAM^+^ and NCAM1^−^EpCAM^+^ cells from BA livers was performed to examine if these cells could generate CLC organoids. NCAM1^+^EpCAM^+^ cell‐derived organoids were of smaller size, unexpanded without a cystic structure. In contrast, the organoids derived from NCAM1^−^EpCAM^+^ cells were relatively larger with cystic structures. Calcein AM staining showed that all these organoids were composed of live cells, indicating that the abnormal morphologic changes in NCAM1^+^EpCAM^+^ organoids were not due to cell apoptosis (Figure 4k). NCAM1^+^EpCAM^+^ and NCAM1^−^EpCAM^+^ organoids were collected for RNA‐seq analysis and the results showed that organoids derived from NCAM1^+^EpCAM^+^ cells had decreased expression of biliary markers (*KRT19* and *HNF1B*; Figure 4l). Transcriptional activity analysis showed that the activities of transcription factors (TFs) essential for biliary lineage (ELF1, STAT1, JUN, GRHL2, SMAD5 and SOX9) were suppressed, while TFs involved in hepatocyte differentiation (NR1I3, HNF4A, SMARCC1, RORA and PPARA) were enhanced, which suggested that NCAM1^+^EpCAM^+^ HPCs were not as effective as the NCAM1^−^EpCAM^+^ HPCs in generating biliary cells (Figure 4m and Supporting Information 1).

### 3.4 NCAM1^+^EpCAM^+^ HPCs Displayed Transcriptomics Associated With Parkinson's Disease and the α‐synuclein Pathway

**FIGURE 5:**
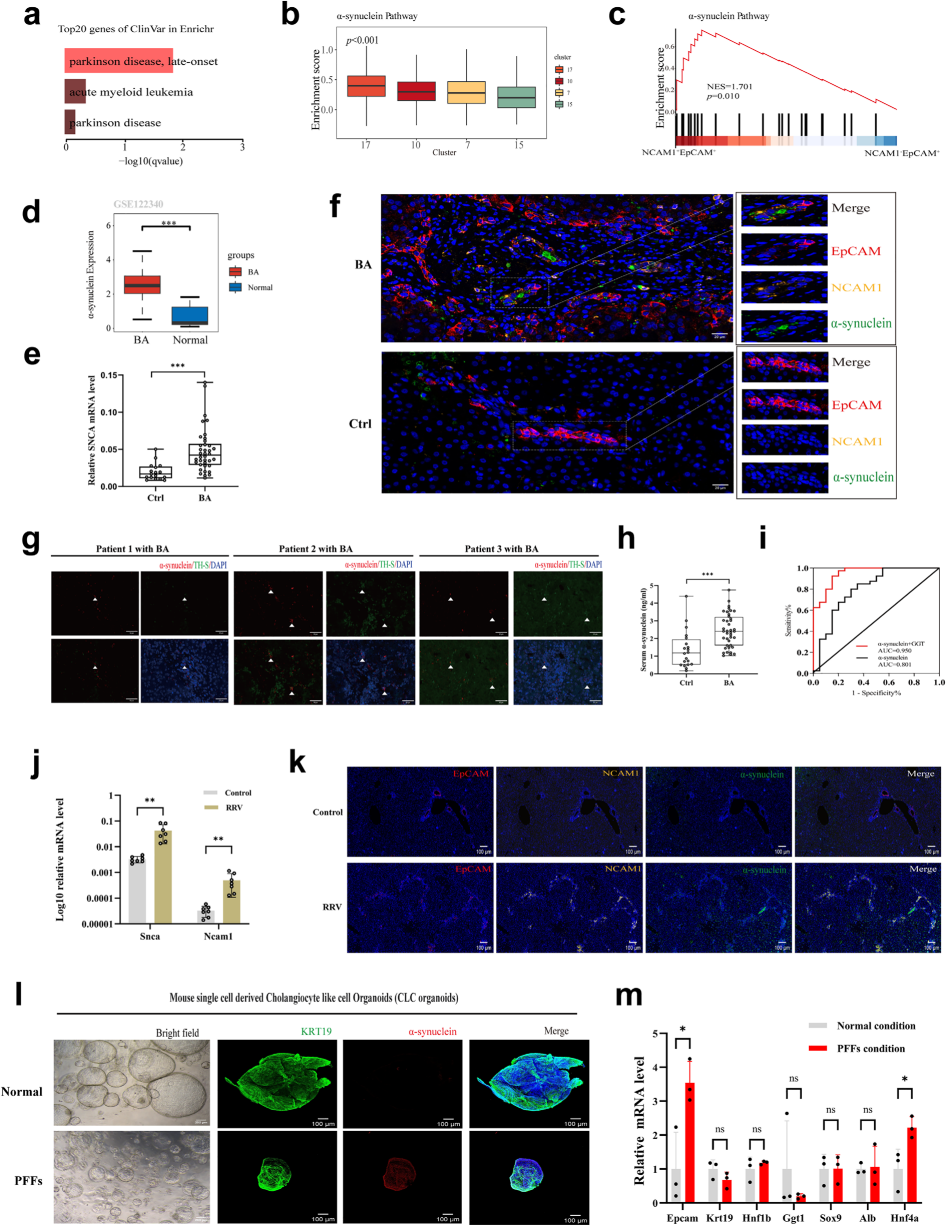
NCAM1^+^EpCAM^+^ HPCs displayed transcriptomics associated with PD and α‐synuclein pathway. (a) Clinical variation analysis of the top 20 genes in cluster 17 calculated by Hypergeometric test. (b) Gene Set Variation Analysis of α‐synuclein pathway in each biliary cluster (cluster 7, 10, 15 and 17). The Kruskal‐Wallis test was used for multi‐group comparisons. (c) Gene Set Enrichment Analysis of α‐synuclein pathway in DEGs from organoids derived from NCAM1^+^EpCAM^+^ and NCAM1^−^EpCAM^+^ cells. *p* value was calculated by Permutation test. (d): The expression of α‐synuclein in BA compared with normal liver tissues in GSE122340 (171 BA and 7 normal liver tissues) calculated by wilcoxon rank‐sum test. (e): The expression of α‐synuclein in BA (*n* = 38) and Ctrl liver tissues (*n* = 17). *P* value was calculated by unpaired t test. (f) Representative immunofluorescence images of NCAM1, EpCAM and α‐synuclein in BA and control sample. Scale bar: 20 µm. (g) Immunofluorescence images of Thioflavin‐S and α‐synuclein co‐staining in 3 patients with BA. The white triangle points to the co‐localization region. Scale bar: 50 µm. (h) Histogram showing serum total α‐synuclein in 40 BAs and 20 controls. *p* value was calculated by unpaired t test. (i) ROC of serum total α‐synuclein combined with or without GGT as a predictor for BA. (j) The mRNA expression of Snca and Ncam1 in RRV (*n* = 7) and Control liver tissues (*n* = 7) after log10 conversion. *P* value was calculated by unpaired t test. (k) Representative immunofluorescence images of NCAM1, EpCAM and α‐synuclein in RRV and control liver tissues. Scale bar: 100 µm. (l) Representative morphology and whole‐mount immunofluorescent staining of KRT19 and α‐synuclein in PFFs‐treated (PFFs) and untreated (Normal) mouse single cell derived CLC organoids. Scale bar of bright field images: 200 µm. Scale bar of immunofluorescent staining image: 100 µm. (m) Relative expression of hepatic and biliary markers in PFFs treated mouse single cell derived CLC organoids (*n* = 3) and normal single cell derived CLC organoids (*n* = 3). *P* value was calculated by unpaired t test. *p < 0.05; **p < 0.01; ***p < 0.001.

Clinical variation analysis of the top 20 DEGs (ranked by log_2_FC) in NCAM1^+^EpCAM^+^ HPCs (cluster 17) indicated that genes including *BACE2*, *SNCAIP*, *NRG3*, *PLXDC2*, and *GAS7* could be linked to Parkinson's disease (PD) (Figure 1f and Figure 5a). Aggregation of α‐synuclein and the loss of dopamine‐producing neurons in the substantia nigra are pathologic hallmarks of PD [16]. GSEA showed that the α‐synuclein pathway enrichment score was significantly higher in cluster 17 compared to other biliary clusters (Figure 5b). Meanwhile, the α‐synuclein pathway was also enriched in organoids derived from NCAM1^+^EpCAM^+^ cells compared to NCAM1^−^EpCAM^+^ cells (Figure 5c). The α‐synuclein expression was significantly higher in BA in both of GSE122340 and our patient cohort (Figure 5d,e). Immunofluorescent staining of liver tissue sections showed an aggregation of α‐synuclein (detected by an antibody specific for the aggregated form of α‐synuclein) around the NCAM1^+^EpCAM^+^ HPCs in BA liver (Figure 5f). Thioflavin‐S and α‐synuclein co‐staining showed the aggregated form of α‐synuclein was partially co‐localized with thioflavin‐S signals (Figure 5g). Serum α‐synuclein has been considered as a marker for a variety of synucleinopathies, including PD. We next determined if α‐synuclein is detectable and is elevated in the serum of patients with BA. In a cohort of patients with 40 BA and 20 controls, the serum total α‐synuclein levels were found to be significantly higher in the BA group (Figure 5h). The Area Under Curve (AUC) for diagnosing BA with serum α‐synuclein alone was 0.801 (95%CI: 0.672‐0.931) with a cutoff of 1.44 ng/mL, while when combined with GGT the AUC reached 0.950 (95%CI: 0.889‐1), suggesting that α‐synuclein could be a potential diagnostic serum biomarker for BA (Figure 5i).

In the Rhesus rotavirus (RRV)‐induced BA mouse models, the mRNA levels of *Snca* and *Ncam1* were significantly upregulated (Figure 5j). Immunofluorescence staining revealed that aggregated α‐synuclein was localized around NCAM1^+^EpCAM^+^ HPCs in the liver of RRV‐induced mice (Figure 5k). When cholangiocyte‐like cell (CLC) organoids derived from mouse hepatic EpCAM^+^ cells were dissociated into single‐cell suspensions and treated with mouse PFFs, the CLC organoids produced were significantly smaller. Meanwhile, immunofluorescence staining confirmed that PFF treatment induced robust upregulation of α‐synuclein in CLC organoids (Figure 5l). qRT‐PCR analysis of PFF‐treated and control CLC organoids showed that the mRNA levels of the hepatocyte marker *Hnf4a* and the HPC marker *Epcam* were significantly elevated, suggesting PFFs could induce aberrant biliary fate (Figure 5m).

### 3.5 α‐synuclein Induced BA Features in iPSC‐Derived CLC Organoids

**FIGURE 6:**
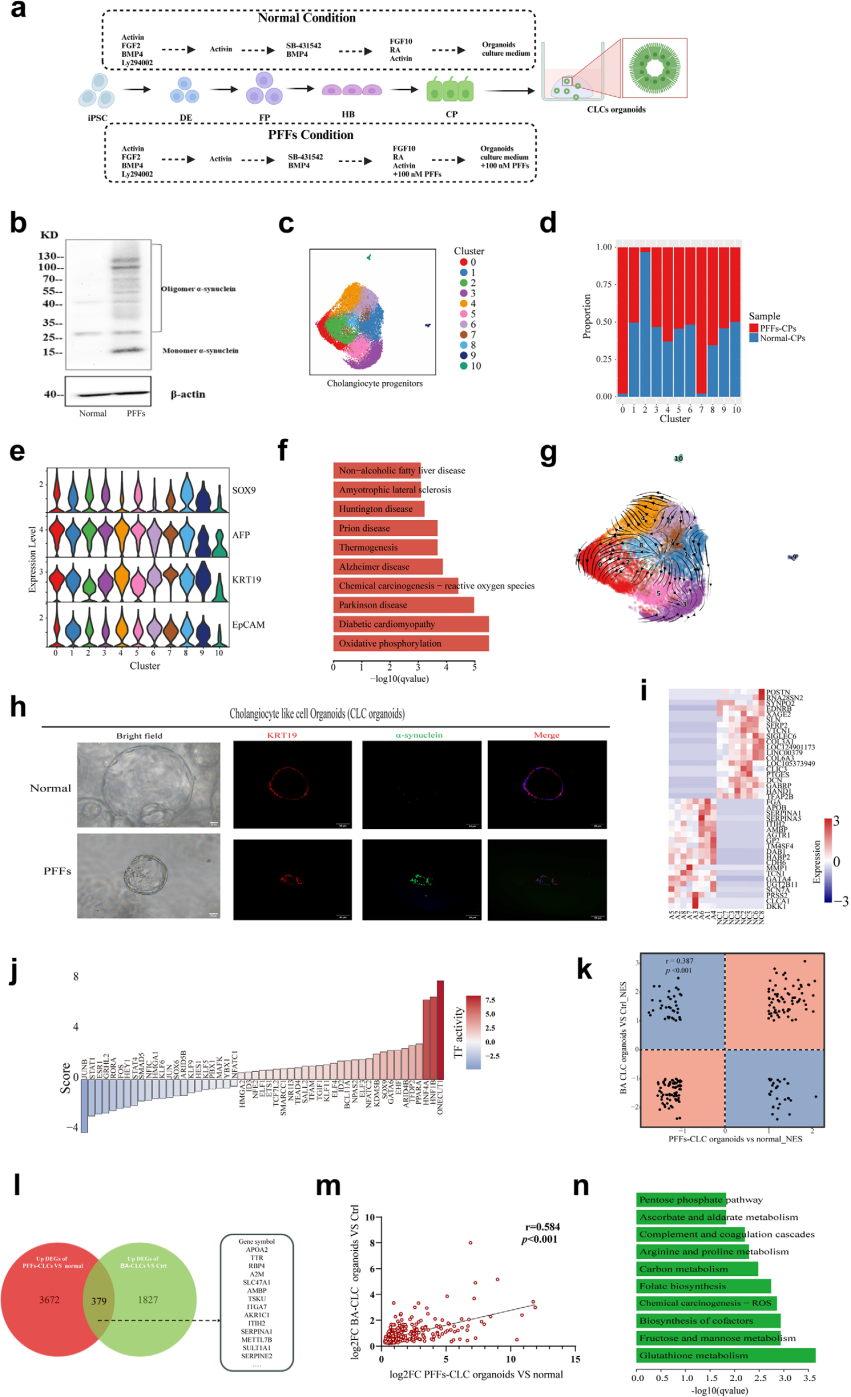
α‐synuclein induced BA features in cholangiocyte‐like cells organoids. (a) Schematic diagram showing the experiment testing the effects of PFFs in the development of iPSC‐derived cholangiocyte‐like cell (CLC) organoids. (b) Western blot analysis for α‐synuclein on PFFs‐treated (PFFs) and untreated (Normal) cholangiocyte progenitors (CPs). (c) UMAP visualization of cells from integrated PFFs‐treated and untreated normal CPs colored according to cluster. (d) The proportion of PFFs‐treated (PFFs‐CPs) and untreated normal CPs (Normal CPs) in each cluster. (e) Violin plot of *SOX9*, *EpCAM*, *AFP* and *KRT19* in each cluster of CPs. (f) KEGG pathways analysis on DEGs of cluster 0 from scRNA‐seq data. The color indicated each cluster and the bar indicates ‐log10(qvalue) of each term. q values represent FDR‐adjusted *p*‐values. *p* value was calculated by Hypergeometric test. (g) RNA velocity analysis of each cluster of CPs by scVelo. (h) Representative morphology and immunofluorescent staining of KRT19 and α‐synuclein in PFFs‐treated (PFFs) and untreated (Normal) CLC organoids. Scale bar of bright field images: 20 µm. Scale bar of immunofluorescent staining image: 50 µm. (i) Heatmap showing up‐ and down‐regulated DEGs of PFFs‐treated (A1‐A8) CLC organoids compared with untreated (NC1‐NC8) CLC organoids. (j) Bar chart showing transcriptional activity of TFs implicated in biliary and hepatic lineage. Red color indicated increased activity, while blue color indicated decreased activity in PFFs‐treated (PFFs) CLC organoids. (k) Pearson correlation analysis of the Normalized Enrichment Score (NES) values of common pathways (NES > 1 or < −1) derived from the differentially expressed genes (DEGs) of two comparative groups: PFFs‐induced CLC organoids versus normal controls and BA liver tissue‐derived CLC organoids versus controls. Each point represents a common pathway. (l) Venn diagram showing the overlapped 379 genes between up‐DEGs (log_2_FC>0.2 and adjusted *p* < 0.05) in PFFs‐treated (PFFs‐CLC organoids) CLC organoids and up‐DEGs (log_2_FC>0.2 and adjusted *p* < 0.05) of EpCAM^+^ cells derived CLC organoids from BA liver tissues (BA‐CLC organoids). (m) Pearson correlation analysis of the log_2_FC value of the overlapped up‐DGEs in PFFs‐treated CLC organoids (PFFs‐CLC organoids) with EpCAM^+^ cells derived CLC organoids from BA liver tissues (BA‐CLC organoids) (*r* = 0.584, *p* < 0.001). (n) Reactome pathway analysis of the overlapped 379 genes. The bar indicated lg(*q*‐value) of each term. *q* values represent FDR‐adjusted *p*‐values. *p*‐value was calculated by Hypergeometric test.

To further elucidate whether α‐synuclein is sufficient to induce aberrant biliary cell development, an iPSC‐derived CLC organoid platform was used. At the beginning of HB differentiation into CLC organoids, human PFFs were added to induce the expression of intracellular α‐synuclein (Figure 6a). Levels of monomeric and oligomeric α‐synuclein were elevated in cholangiocyte progenitors (CPs) derived from the PFFs‐treated HBs compared to the untreated HBs (Figure 6b). However, flow cytometry analysis indicated that CPs were formed in PFFs‐treated and untreated cultures (Figure S3a). To further investigate the cellular effects of α‐synuclein on CPs development, single‐cell RNA sequencing was performed on CPs in PFFs‐treated and untreated cultures. CPs were clustered into 11 populations; cluster 0 of CPs accounted for the highest proportion of PFFs‐CPs (Figure 6c,d and Figure S3b). All the clusters expressed biliary markers (*KRT19* and *SOX9*; Figure 6e), which was consistent with flow cytometry analysis of CPs formed from both PPFs‐treated and ‐untreated HBs. Kyoto Encyclopedia of Genes and Genomes (KEGG) analysis of the highly expressed DEGs of cluster 0 of CPs revealed a significant enrichment in pathways associated with various neurodegenerative disorders, with the most enriched pathway related to oxidative stress (Figure 6f and Figure S3c). Trajectory analysis showed that CPs transitioned toward cluster 0 following treatment with PFFs (Figure 6g).

CLC organoids derived from PFFs‐treated CPs exhibited a vacuolated morphology with smaller sizes and unclear boundaries, and accumulation of α‐synuclein (Figure 6h). Furthermore, organoids from PFFs‐treated and ‐untreated CPs were picked for RNA sequencing. Principal component analysis (PCA) revealed significant transcriptomic differences between PFFs‐treated CLC organoids (PFFs‐CLC organoids) and untreated controls (Figure S3d). The DEGs heatmap showed that upregulated DEGs of PFFs‐CLC organoids were associated with hepatocyte differentiation, including *FGA*, *SERPINE1*, *MMP1*, and *DKK1* (Figure 6i). TF activity based on PFFs‐CLC organoids DEGs showed that although classical biliary fate determination factors (ONECUT1, SOX9, and HNF1B) were activated, activities of TFs contributing to hepatocyte differentiation (HNF4A, PPARA, and ARID4B) were also increased (Figure 6j). Meanwhile, GSEA of DR related pathways (Hippo, TGF, WNT, Hedgehog, and Notch) on DEGs between PFFs‐CLC organoids and untreated controls showed only hedgehog signaling pathway was negatively and significantly enriched (adjusted *p* < 0.001, NES = ‐1.957; Figure S3e).

Previous study showed that organoids derived from EpCAM^+^ cells in BA liver tissues also exhibit unstable biliary fate [10]. To investigate the association between PFFs‐ CLC organoids and BA liver tissue‐derived CLC organoids, the DEGs from two comparative groups: PFFs‐CLC organoids versus normal and BA EpCAM^+^ cell‐derived CLC organoids versus controls, were obtained. GSEA of pathways from Wiki‐pathway data set was performed separately on the DEGs of two comparative groups. Pearson correlation analysis of the Normalized Enrichment Score (NES) of these shared pathways across both comparative groups revealed a significant positive correlation (r = 0.387, *p* < 0.001) (Figure 6k). Moreover, intersection of up‐regulated DEGs (log_2_FC>0.2 and adjusted *p* < 0.05) in PFFs‐CLC organoids with DEGs (log_2_FC>0.2 and adjusted *p*< 0.05) of EpCAM^+^ cell‐derived CLC organoids from BA liver tissues showed that 379 genes overlapped and the log_2_FC values of these 379 genes had a significant positive correlation between the two datasets (r = 0.584, *p* < 0.001; Figure 6l, m and Supporting Information 1). Pathway enrichment analysis of these shared DEGs identified significant enrichment in multiple metabolic pathways with the glutathione (GSH) metabolic pathway exhibiting the greatest statistical significance (Figure 6n, GO and pathways annotation of DEGs of both comparative groups presented in Figure S3f,g).

### 3.6 α‐synuclein Enhanced the Susceptibility of Biliary Epithelial Cells to GSH Redox Imbalance

**FIGURE 7:**
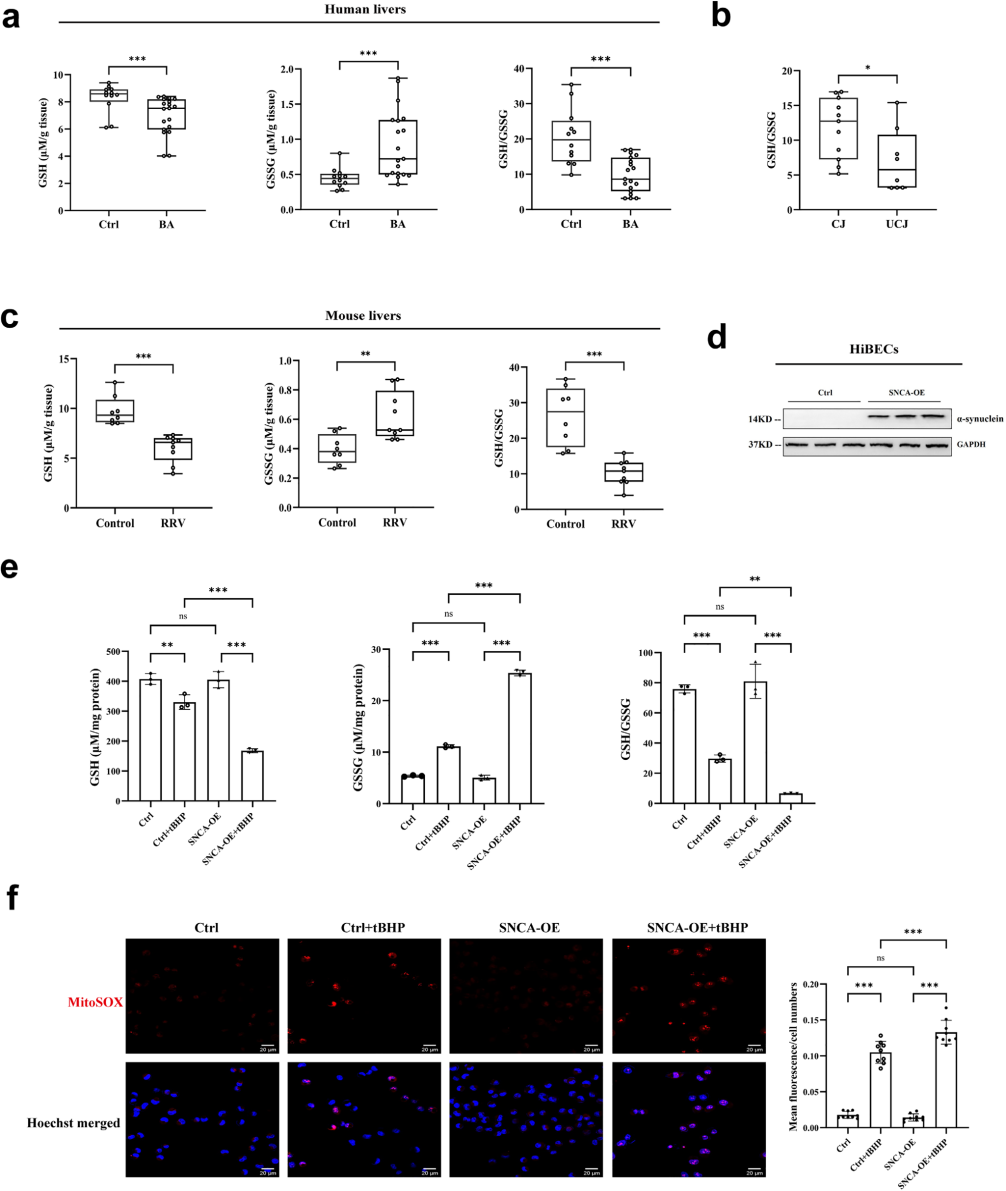
α‐synuclein enhanced the susceptibility of biliary epithelial cells to GSH redox imbalance. (a) From left to right: comparisons of GSH content, GSSG level, and GSH/GSSG ratio between BA liver tissues (*n* = 19) and control liver tissues (*n* = 12). *P* value was calculated by unpaired t test. (b) Comparison of the GSH/GSSG ratio between the successful clearance of jaundice (CJ) group (*n* = 11) and the unsuccessful clearance of jaundice (UCJ) group (*n* = 8). *P* value was calculated by unpaired t test. (c) From left to right: comparisons of GSH content, GSSG level, and GSH/GSSG ratio between RRV liver tissues (*n* = 9) and control liver tissues (*n* = 8). *P* value was calculated by unpaired t test. (d) Western blot analysis for α‐synuclein on α‐synuclein‐overexpressing and control human intrahepatic biliary epithelial cells (HiBECs). (e) Panels are arranged from left to right to show comparisons of GSH content, GSSG level, and the GSH/GSSG ratio in four groups: control group (Ctrl), control group treated with 5 µM tert‐butyl hydroperoxide (tBHP) for 24 h (Ctrl + tBHP), *SNCA*‐overexpressing group (SNCA‐OE), and *SNCA*‐overexpressing group treated with tBHP for 24 h (SNCA‐OE + tBHP). *P* value was calculated by two‐way ANOVA followed by Tukey's post hoc test; *n* = 3 per group. (f) Representative immunofluorescent staining of MitoSOX red and Hoechst 33342 in Ctrl, control group treated with 5 µM tert‐butyl hydroperoxide (tBHP) for 24 h (Ctrl + tBHP), SNCA‐OE, and *SNCA*‐overexpressing group treated with tBHP for 24 h (SNCA‐OE + tBHP). Scale bar: 20 µm. MitoSOX Red fluorescence was detected by confocal microscope. Hoechst 33342 staining was performed to quantify total cell numbers. *P* value was calculated by two‐way ANOVA followed by Tukey's post hoc test; *n* = 9 per group. *p < 0.05; **p < 0.01; ***p < 0.001, ns: not significant.

Total cellular glutathione comprises two forms: reduced glutathione (GSH), and oxidized glutathione disulfide (GSSG) — the latter formed via dehydrogenation of thiol groups from two GSH molecules. We first measured the content of GSH and GSSG in human BA and control liver tissues (Ctrl), and found that BA's liver exhibited a significant reduction in GSH content, but a significant increase in GSSG levels, and a significantly decreased GSH/GSSG ratio (Figure 7a). Furthermore, we found that the GSH/GSSG ratio was significantly higher in BA patients with successful clearance of jaundice (CJ) than in those with unsuccessful clearance of jaundice (UCJ) (Figure 7b). In RRV‐induced BA mouse models, hepatic GSH content was also significantly decreased, GSSG level was significantly increased, and the GSH/GSSG ratio was significantly reduced (Figure 7c), suggesting that disrupted GSH redox homeostasis is a conserved feature of BA pathogenesis.

To further clarify the effect of α‐synuclein on GSH metabolism, we generated lentiviral transduced human intrahepatic biliary epithelial cells (HiBECs) that stably overexpressed *SNCA* (SNCA‐OE), alongside with empty vector‐transduced control cells (Ctrl) (Figure 7d). We found that there were no significant differences in GSH/GSSG level, or the GSH/GSSG ratio between SNCA‐OE and Ctrl cells. However, following exposure to the oxidative stress inducer tert‐butyl hydroperoxide (t‐BHP), the SNCA‐OE group exhibited significantly lower GSH content and GSH/GSSG ratio, alongside a markedly elevated GSSG level, relative to the Ctrl group (Figure 7e). Measurement of mitochondrial ROS levels also showed that *SNCA* overexpression alone did not increase mitochondrial ROS abundance. However, upon challenge with t‐BHP, cells in SNCA‐OE group exhibited significantly higher mitochondrial ROS levels relative to Ctrl (Figure 7f). Taken together, these findings indicated that elevated α‐synuclein expression increases the susceptibility of HiBECs to GSH‐associated redox imbalance under oxidative stress.

## 4 Discussion

BA is a severe cholestatic liver disease which is characterized by the involvement of both intra‐ and extra‐hepatic bile ducts. Currently, Kasai surgery is the first‐line treatment but the long‐term prognosis remains poor [17]. Moreover, Kasai surgery only replaces the extrahepatic bile ducts and intrahepatic cholangiopathy is unremedied, leading to liver failure requiring liver transplantation. A thorough understanding of the aetiologic mechanisms underlying the pathophysiologic intrahepatic bile duct development in BA is critical for developing diagnostic markers and therapeutic strategies. In the current study, snRNA‐seq on BA and non‐BA livers identified a significant expansion of a cell population in BA with characteristics of EpCAM^+^ HPCs. Specifically, these EpCAM^+^ HPCs in BA had the following characteristics: (i) high NCAM1 expression; (ii) impaired biliary organoid growth and disruption of biliary lineage determination; and (iii) features associated with PD and the α‐synuclein pathway. Deposition of α‐synuclein was detected at and around the NCAM1^+^EpCAM^+^ HPCs in BA livers and serum α‐synuclein was elevated in patients with BA. Intracellular accumulation of α‐synuclein in cholangiocyte progenitors induced defective BA‐like biliary development in iPSC‐derived CLC organoids.

The DR is one of the most significant histopathologic features of BA. The DR mainly consists of hyperproliferative or reactive cholangiocytes, dedifferentiated hepatocytes, and HPCs. Parenchymal cells, including cholangiocytes, hepatocytes, and HPCs, interact with the surrounding immune cells, endothelial cells, and fibroblasts in biliary injury, forming distinct niches for biliary repair and biliary regeneration [18]. HPCs are stem cell‐like with bidirectional differentiation potentials. In response to injury, HPCs proliferate and differentiate into hepatocytes and cholangiocytes, thereby contributing to liver regeneration in damaged liver tissue [19, 20]. HPCs exhibit diverse molecular markers across different animal models and liver pathologies, including OV6, LGR5, TROP2, CD24, CD133, EpCAM, and SOX9, with EpCAM currently serving as the predominant marker for HPC identification [14, 21]. Integrated scRNA‐seq and spatial transcriptomic analyses of human liver tissues have revealed that EpCAM‐high HPCs compartmentalize into three functionally divergent subpopulations, providing evidence of intrinsic heterogeneity within the HPC pool [22]. A human liver cell atlas has revealed that EpCAM^+^Trop2^int^ HPCs in human liver have enhanced capacity to generate bipotent liver organoids, suggesting potential functional significance in hepatic regeneration processes [23]. However, the above studies suggest that additional markers, in addition to EpCAM, are needed to delineate HPC subsets in different disease contexts.

scRNA‐seq and spatial transcriptomics of immune cell atlases and fibrosis niches in human BA liver have been reported [24, 25]. However, these studies have not fully elucidated the characteristics of the intrahepatic bile ducts in BA. Because snRNA‐seq favorably captures the landscapes of hepatic parenchymal cells [26], snRNA‐seq was performed on BA and non‐BA livers in this study, we identified a cell population (cluster 17) that expressed high EpCAM and NCAM1 levels and was uniquely expanded in BA. The expanded cell population was described as NCAM1^+^EpCAM^+^ HPCs because the cells exhibited typical HPC features, including transcriptomic signatures enriched for BA‐associated DR‐related repair and regeneration pathways. NCAM1^+^EpCAM^+^ HPCs interact with other cell types (immune, endothelial, and mesenchymal cells), in particular, with the strongest interactions with fibroblasts/stellate cells. Fibroblasts/stellate cells produce signaling molecules that participate in biliary repair and regeneration [27]. Laminin and collagen families were involved in the interaction between NCAM1^+^EpCAM^+^ HPCs and fibroblasts/stellate cells. Laminin and collagen families are main members involved in ECM remodeling and matrix deposition in HPCs‐mediated liver regeneration [28]. Intriguingly, the interaction strength analysis revealed a decrease in the interaction between macrophages and NCAM1^+^EpCAM^+^ HPCs in BA compared to the Ctrl1/Ctrl2 group, which may reflect macrophage dysfunction, as previously reported in BA [24].

NCAM1^+^EpCAM^+^ HPCs have previously been reported to reside in the ductular plates (DPs) of human fetal liver and in the canals of Herring of normal adult liver [29]. Notably, transplantation studies demonstrated that NCAM1^+^EpCAM^+^ HPCs exhibit renal capsule engraftment competency, expanding into fumarylacetoacetate hydrolase‐ and HNF4A‐expressing hepatocytes, whereas the NCAM1^−^EpCAM^+^ cells showed impaired regenerative potential in this ectopic microenvironment [30]. Previous studies have demonstrated that NCAM1^+^EpCAM^+^ HPCs are involved in liver repair/regeneration in physiologic conditions but their roles in pathologic repair/regeneration during ductular injury in BA remain unclear. Dysregulated proliferation and differentiation of HPCs leads to an accumulation of HPCs in liver, which not only hinders liver regeneration but also promotes liver deterioration and disease progression [18, 31]. Therefore, the proper differentiation of HPCs in response to injury is crucial for liver regeneration, including the bile ducts. These NCAM1^+^EpCAM^+^ HPCs exhibited impaired biliary development with a reduced expression of biliary markers. Reduced activity of the TFs for biliary lineage but an enhanced activity of TFs for hepatocyte lineage was observed in NCAM1^+^EpCAM^+^ biliary organoids. NCAM1 has been reported to be highly expressed in BA. NCAM1‐positive bile ducts present with more bile plugs, which suggest severe bile duct obstruction [32]. Taken together, these findings suggest that NCAM1^+^EpCAM^+^ HPCs in BA liver have a biliary differentiation defect and abnormal biliary development of NCAM1^+^EpCAM^+^ HPCs may generate abnormal bile ducts localized at the DR regions in BA liver sections.

DEGs analysis revealed that NCAM1^+^EpCAM^+^ HPCs are associated with PD with α‐synuclein, which was also localized around NCAM1^+^EpCAM^+^ cells in liver tissue sections of BA. PFFs‐treated CLC organoids exhibited typical features of BA tissue‐derived CLC organoids. α‐synuclein exists in phosphorylated and oligomeric forms in synucleinopathies with the oligomers existing in both soluble and insoluble forms [33]. Although oligomers of α‐synuclein were detected in PFF‐treated organoids, whether α‐synuclein exists in aggregates of monomeric / oligomeric / polymeric forms in BA liver and whether the aggregation of α‐synuclein is associated with disease progression are needed to be determined. Beta‐amyloid (Aβ) accumulation is noted around bile ducts in BA livers and exposure to Aβ induces aberrant morphology in control organoids [10]. Both α‐synuclein and Aβ hybrid oligomers have been detected in brain tissues of patients with Alzheimer's disease (AD) and PD [34]. Moreover, both monomeric and oligomeric α‐synuclein act as a seed and facilitate Aβ oligomerization and inhibit fibrillation [35], while Aβ42 induces α‐synuclein aggregation in neurons [36]. Aβ also interacts indirectly with α‐synuclein by inducing phosphorylation of α‐synuclein [37]. Both Aβ and α‐synuclein (current study) are localized at abnormal bile ducts in the DR regions of BA livers; it remains to be determined if these two proteins accumulate at the same time or in a sequential order, and if hybrid aggregates are formed in BA livers. While both Aβ and α‐synuclein (current study) induce the observed abnormal morphologic changes in organoids, it remains to be determined whether these protein accumulations are causative or bystander effects resulting from other pathogenic mechanisms in BA.

Circulating α‐synuclein has been shown to serve as an early biomarker for PD but with a poor sensitivity of 71% and specificity of 64% [38]. The diagnostic efficacy of the circulating total α‐synuclein was relatively low (approximately 0.8) in the BA cohort, but when combined with the GGT level, the diagnostic accuracy improved to approximately 0.95. Considering that α‐synuclein has different forms, it remains unclear whether the combination of various forms of α‐synuclein‐related biomarkers can improve the diagnostic performance for BA. Plasma Aβ42/Aβ40 are elevated in BA and the combinations of plasma Aβ42/Aβ40, GGT and another hepatic function parameter could differentiate BA from choledochal cyst with cholestasis [39]. Whether combining Aβ with α‐synuclein plus GGT could further enhance the early diagnostic accuracy for BA requires further investigation. The seed amplification assay, which exploits the self‐replication of misfolded α‐synuclein through fragmentation and elongation cycles, has emerged as a promising tool for early diagnosis [40]. Whether this method is applicable for early detection of BA warrants further investigation.

Considering the isolation of HPCs from normal human liver tissue for organoid culture is limited by factors such as ethical constraints, donor availability, and culture efficiency, rendering it not highly feasible [41]. HBs and HPCs are bipotent liver cells identified during early and late embryonic development, respectively [42]. The process of biliary regeneration following liver injury is consistent with that of embryonic biliary development [3, 43]. Functional similarities between HBs and HPCs prompted the use of an iPSC biliary organoid differentiation model to investigate the effects of α‐synuclein in biliary development. PFF‐treated iPSC derived CLC organoids and BA liver‐derived CLC organoids share a morphologic congruence as well as transcriptomic convergence on GSH metabolic dysregulation. BA with successful clearance of jaundice or prolonged native liver survival are associated with activated GSH metabolism, while the administration of N‐acetylcysteine to improve GSH metabolism or direct supplementation of GSH significantly reduces liver injury and fibrosis in experimental BA mouse models, suggesting that the perturbed GSH metabolic pathway is a potential therapeutic target for BA [44, 45, 46]. The accumulation of α‐synuclein aggregates can lead to mitochondrial dysfunction and subsequent GSH depletion [47, 48]. Meanwhile, mitochondrial oxidative stress or altered GSH metabolism could promote α‐synuclein aggregation, creating a vicious cycle in PD [49, 50]. During the induction of iPSC‐derived CLC organoids with BA features by PFFs, Hedgehog signaling pathway was found to be aberrantly suppressed. The Hedgehog signaling pathway is widely recognized to play an essential role in liver organogenesis and hepatic tissue repair [51, 52]. During these processes, the normal activation and maintenance of the Hedgehog signaling pathway are highly dependent on primary cilia [53]. As reported, the accumulation of α‐synuclein impairs ciliary function in neurons, thereby compromising the cellular response to oxidative stress [54]. Concurrently, the occurrence and progression of BA have been linked to cilia‐related gene mutations and ciliary dysfunction [55, 56]. Therefore, we hypothesize that α‐synuclein aggregation may disrupt the function of primary cilia, subsequently leading to elevated intracellular oxidative stress levels and the inhibition of Hedgehog signaling, which ultimately impairs biliary regeneration. However, this hypothesis still requires further experimental validation in future studies.

Despite the identification of α‐synuclein as a factor influencing HBs‐biliary differentiation in BA, the current study was not without limitations. First, the DR features of biliary clusters were elucidated through bioinformatics. Co‐culture of DR‐related HPCs with periportal immune, endothelial, and/or mesenchymal cells to clarify changes in their respective cellular phenotypes and the accuracy of ligand‐receptor interactions would strengthen the evidence for the roles of HPCs in the DR process in BA. Second, the association between α‐synuclein aggregation and HPCs in BA was inferred from bioinformatics annotations, although we confirmed the presence of α‐synuclein aggregation in BA livers via fluorescent staining. The aggregation of α‐synuclein around HPCs in BA does not imply the same etiology as PD, and the specific mechanisms require further investigation. Last but not least, the sample size for snRNA‐seq was relatively small, which may introduce bias into our understanding of the comprehensive landscape of HPCs in BA, and integrating a larger cohort data to include prognostic information could provide valuable insights into the role of α‐synuclein‐associated HPCs in the pathogenesis and progression of BA.

In conclusion, the current data revealed the following: (i) enrichment of a NCAM1^+^EpCAM^+^ subpopulation in DR‐associated HPCs in BA; (ii) NCAM1^+^EpCAM^+^ HPCs displayed aberrant biliary differentiation; (iii) NCAM1^+^EpCAM^+^ HPCs‐derived biliary organoids upregulated genes enriched in an α‐synuclein pathway and PD and α‐synuclein was detected to be aggregated around NCAM1^+^EpCAM^+^ HPCs in BA livers; (iv) α‐synuclein induced BA‐like features in biliary organoids. This protein disrupts the proper differentiation of HPCs into the biliary lineage, resulting in impaired biliary regeneration. The current study has provided new insights into the pathophysiologic characteristics of intrahepatic bile ducts and offers potential target for biliary repair and regeneration for BA. Furthermore, targeting α‐synuclein represents a potential therapeutic strategy in synucleinopathies, including PD. However, given that targeting α‐synuclein remains a preclinical hypothesis requiring substantial further research for clinical translation, it is yet unknown whether this strategy can be clinically applied as an adjuvant therapy for BA.

## Author Contributions

Q.W., V.C.H.L., Y.X., and W.T. conceived the study. H.X. and Z.Z. designed the study and wrote the original draft. H.X., M.Z., and J.T. contributed to the analysis and visualization of the sequencing data. R.Z. and Z.D. collected clinical samples and analyzed the clinical data. J.L. and Z.W. conducted liver tissue and iPSC derived organoids cultures. H.X., M.Z., and V.C.H.L. verified the underlying data. P.K.H.T., V.C.H.L., and W.T. revised and edited the manuscript. All the authors have read and approved the final manuscript.

## Funding

This study was financially supported by the National Natural Science Foundation of China (Grant No. 82370523) to WB Tang,  the National Natural Science Foundation of China (Grant No. 82221005) to YK Xia, and the Theme‐based Research Scheme 2021 (Grant No. T12‐712/21‐R) to VCHL.

## Conflicts of Interest

The authors declare no conflicts of interest.

## Supporting information




**Supporting File 1**: advs76054‐sup‐0001‐SuppMat.docx.

**Supporting File 2**: advs76054‐sup‐0002‐SuppMat.docx.

**Supporting File 3**: advs76054‐sup‐0003‐TablesS1‐S7.docx.

**Supporting File 4**: advs76054‐sup‐0004‐Data.xlsx.

**Supporting File 5**: advs76054‐sup‐0005‐Data.pdf.

## Data Availability

The data that support the findings of this study are available from the corresponding author upon reasonable request.
