# Supplementary material for: Expanded Hepatic Progenitor Cells Featured with Aggregation of α‐Synuclein Contribute to Pathologic Bile Duct Regeneration in Biliary Atresia
Source: Adv Sci (Weinh). 2026 Jun 29;13(50):e76054. doi: 10.1002/advs.76054 (PMC13336565; doi:10.1002/advs.76054)
Supplement: Supplementary file 4 [file ADVS-13-e76054-s004.docx]

**Supplemental methods**

**Single nucleus isolation and sequencing procedures**

Nuclei were isolated using a Shbio Nuclei Isolation kit (#52009-10; China). Briefly, the frozen tissue samples were quickly added to 1 mL of lysis buffer and the tissue was homogenized to a liquid state without tissue blocks using a tissue homogenizer. The tissue lysate was passed through a 40-μm cell strainer (Falcon, USA) to remove tissue clumps, collected into a 2-mL EP tube, and centrifuged at 500 g for 5 min at 4°C to collect the nuclei pellet. Next, PB1, PB2, and PB3 solutions (#52009-10; China) were added to the pellet and the nuclei were collected at the junction of PB2 and PB3 solutions. Finally, the nuclei were resuspended in 50 μL of NB solution (#52009-10; China). The nuclei were counted with a cell counter (ThermoFisher Scientific). Using a Chromium Single Cell 3′ Library and Gel Bead kit [v3] (10X Genomics, USA), nuclei were immediately loaded onto a Chromium Single Cell Processor (10X Genomics) for barcoding of RNA from single nuclei. Sequencing libraries were constructed according to the manufacturer’s instructions (10X Genomics), then sequenced using a NovaSeq 6000 sequencing system (Illumina, USA).

**10× library preparation and sequencing**

Beads with a unique molecular identifier (UMI) and cell barcodes were loaded close to saturation so that each cell was paired with a bead in a Gel Beads-in-emulsion (GEM). After exposure to cell lysis buffer, polyadenylated RNA molecules were hybridized to the beads. Beads were retrieved into a single tube for reverse transcription. Each cDNA molecule was tagged on the 5’end (i.e., the 3’end of a messenger RNA transcript) with UMI and cell label indicating the cell of origin. Briefly, 10× beads were then subjected to second-strand cDNA synthesis, adaptor ligation, and universal amplification. Sequencing libraries were prepared using randomly interrupted whole-transcriptome amplification products to enrich the 3’ end of the transcripts linked with the cell barcode and UMI. All the remaining procedures, including the library construction, were performed according to the standard manufacturer’s protocol (CG000206; RevD, USA). Sequencing libraries were quantified using a High Sensitivity DNA Chip (Agilent, USA) on a Bioanalyzer 2100 using the Qubit High Sensitivity DNA Assay (ThermoFisher Scientific). The libraries were sequenced on a NovaSeq6000 (Illumina) using 2x150 chemistry.

**snRNA-seq and scRNA-seq pre-processing**

Raw sequencing reads were processed using the Cell Ranger pipeline to generate gene expression profiles. The --include-introns parameter was additionally used for snRNA-seq data to retain intronic region information. Ambient RNA molecules were then removed from the snRNA-seq data using the CellBender algorithm^1^ with the total-droplets-included parameter set to 30,000 and the fpr parameter set to 0.01 to ensure high-confidence cell capture. The filtered expression matrix was then loaded using the Seurat package^2^. For downstream analysis, genes expressed in < 3 cells and cells expressing < 200 genes were excluded. Additionally, stringent cell filtering criteria were applied, retaining only cells expressing between 500 and 5000 genes with expression levels > 500 reads but not > 10,000 reads. Cells with mitochondrial transcript content > 1% of total transcripts or ribosomal transcript content > 5% of total transcripts were also excluded.

The cleaned Seurat objects were then subjected to a standard analytical workflow, which included data normalization, highly variable gene detection, data scaling, principal component analysis (PCA), unsupervised clustering, and visualization using Uniform Manifold Approximation and Projection (UMAP). To further mitigate potential technical noise and batch effects, multiple Seurat objects were integrated using the BBKNN algorithm within the Scanpy framework.

The plot density function from the Nebulosa package was used to visualize the density distribution of target genes in the UMAP space, which revealed localized enrichment patterns of gene expression. Additionally, the scANVI algorithm was used to transfer annotated cell type labels to a new dataset^3^. By integrating the reference and new datasets, scANVI leverages variational inference to learn shared features, which enables cross-dataset cell type annotation while mitigating batch effects^4^.

**Differential abundance analysis**

Differential abundance of clusters between groups was assessed using Case-Control Analysis (CACOA) to identify BA-associated cell subpopulations. Cluster-specific enrichment results were further validated by directly comparing inter-group or inter-sample cluster proportions.

**Functional enrichment analysis**

Gene set enrichment analysis (GSEA) and functional enrichment analysis were performed using the clusterProfiler package. The AddModuleScore function from the Seurat package was utilized to quantify pathway activity at the single-cell level in scRNA-seq data. Pathway activity was assessed using the single-sample gene set enrichment analysis (ssGSEA) algorithm implemented in the GSVA package for bulk RNA-seq or microarray data, which calculated gene set enrichment scores for each individual sample.

**Stemness estimation**

CytoTRACE, a computational methodology, was designed to quantify the relative differentiation status of individual cells based on scRNA-seq datasets^5^. This algorithm can predict the trajectory of cellular differentiation autonomously without reliance on preliminary directional information. Input data for this analysis comprises a count matrix, which is extracted from single-cell transcriptomic profiles. The output is a continuum ranging from 0, signifying a greater degree of cellular differentiation, to 1, which denotes a lesser degree of differentiation.

**RNA velocity**

RNA velocity analysis was performed using scVelo, starting with extracting spliced and unspliced matrices from Cell Ranger BAM files via the velocity tool. Then, these matrices were imported into scVelo and the dynamic mode was applied to infer transcriptional dynamics and latent time.

**Cell-cell communication**

Intercellular communication networks were systematically interrogated using CellChat, a computational framework leveraging curated ligand-receptor interaction databases to infer signaling pathways.

**Smart-seq2 data analysis**

Bulk RNA-seq data generated by Smart-seq2 were processed using RSEM for transcript quantification. The reference genome was prepared using rsem-prepare-reference with the STAR aligner, incorporating the human reference genome (GRCh38) and corresponding annotation. Raw reads were aligned to the reference using STAR, followed by expression quantification via rsem-calculate-expression to generate raw read counts and transcripts per million (TPM) values. Gene-level count matrices from RSEM were imported into R using tximport to preserve transcript-to-gene summarization and account for transcript length biases^6^. Differential expression analysis was performed using DESeq2.

**Transcription activity inference**

TF activity was inferred from bulk transcriptomic data generated by Smart-seq2 using the decoupleR algorithm^7^. Raw expression matrices were normalized using transcripts per million (TPM) quantification. TF-target regulatory networks were obtained from the Dorothea database (https://saezlab.github.io/dorothea/), a comprehensive resource of curated TF-gene interactions. Activity scores for each TF were calculated using a univariate linear model (ulm) within decoupleR, which enabled robust estimation of TF activity levels across the samples.

**Rhesus rotavirus mouse model**

Rhesus rotavirus (RRV) strain mmu18006 was generously provided by Dr. Jiexiong Feng (Department of Pediatric Surgery at Tongji Hospital, China). RRV induced BA mice models were constracted as we reported previously^8^. Briefly, neonatal BALB/c mice were injected intraperitoneally with 20 μL of the RRV suspension within 24 h of birth. Controls received the same volume of saline. Mice were monitored each day for weight reduction and jaundice. At 14 days post-injection, liver tissues of both groups were collected to isolate RNA and histological analysis.

**Cell culture, Lentiviral infection and Tert-butyl hydroperoxide treatment**

Human intrahepatic biliary epithelial cells (HiBECs) were purchased from the Shanghai Fuyu Biotechnology Co (Shanghai, China) and cultured under the condition of 37 ℃ and 5 % CO_2_ in DMEM (C11995500BT, Gibco, Grand Island, NY), supplemented with 10 % fetal bovine serum (FBS), 1 % penicillin/streptomycin. Lentiviruses containing human α-synuclein (SNCA) and the scramble control for human were purchased from GenePharma (Shanghai, China). L[entivirus](https://www.sciencedirect.com/topics/pharmacology-toxicology-and-pharmaceutical-science/lentivirus) and 5 mg/mL [polybrene](https://www.sciencedirect.com/topics/pharmacology-toxicology-and-pharmaceutical-science/hexadimethrine-bromide) (GenePharma) was incubated when cells were fused to 40 %. After 48 h, cells were treated with 1 μg/mL [puromycin](https://www.sciencedirect.com/topics/pharmacology-toxicology-and-pharmaceutical-science/puromycin) (ST551, Beyotime, China) to select the infection positive cells. The mRNA and protein levels of SNCA were determined by qRT-PCR and western blot, respectively, to verify the stable overexpression of α-synuclein in the cell lines. Prior to the measurement of cellular GSH and mitochondrial ROS, α-synuclein-overexpressing and control cells were treated with 5 μM tert-butyl hydroperoxide (t-BHP) ([B802372](https://www.macklin.cn/products/B802372), Macklin, China) for 24 h, respectively.

**Measurement of GSH and Mitochondrial ROS levels**

Liver tissues of human BA, mice RRV models or the corresponding controls were weighted, then homogenized, and cells were lysed simultaneously. α-synuclein overexpressed and control HiBECs with or without 5 μM t-BHP treatment were collected. For each cell group, half of the cell samples were used for total protein content determination via the bicinchoninic acid (BCA) assay, while the remaining half was lysed for GSH measurement. The lysates of liver tissues and cells were centrifuged at 10,000× g for 10 min, and the supernatants were collected. GSH and GSSG contents in the supernatant of tissues and cells were detected using the GSH and GSSG AssayKit (S0053, Beyotime, China) according to the manufacturer’s protocol. Finally, the measured contents of GSH and GSSG were normalized by dividing by the weight of liver tissue or the total protein amount of cells, respectively. Mitochondrial ROS levels were detected using the MitoSOX™ Red Mitochondrial Superoxide Indicator (M36008, Thermo Fisher Scientific). Briefly, MitoSOX Red stock solution was prepared by dissolving the reagent in DMSO to a final concentration of 5 mM, which was further diluted in pre-warmed cell culture medium to a working concentration of 5 μM prior to use. α-synuclein overexpressed and control HiBECs with or without 5 μM t-BHP treatment were seeded in the confocal dish and incubated with the MitoSOX Red working solution at 37 °C under dark conditions for 30 min, followed by two washes with D-PBS. After hoechst 33342 (C1022, Beyotime, China) was added, the fluorescence intensity was subsequently determined using a confocal microscope (Leica laser) at an excitation wavelength of 510 nm and an emission wavelength of 580 nm.

**Western blot**

Cell Lysis Buffer (9803S; Cell Signaling Technology, USA) was used to extract total proteins of cholangiocyte progenitors from the normal and PFFs induced group or α-synuclein-overexpressing and control cells. Protein concentrations were determined using BCA Protein Assay Reagent (TaKaRa; Japan). Equal amounts of proteins (50 µg) were separated by electrophoresis on a 12.5% sodium dodecyl sulfate polyacrylamide gel and semi-dry transferred to polyvinylidene difluoride membranes (1.5A, 600 s). After blocking with 5% fat-free milk, membranes were incubated with anti-α-synuclein primary antibodies at 4°C overnight. After washing, the membranes were incubated with a secondary antibody at 25℃ for 2 h. Signals were developed using an enhanced chemiluminescence reagent following the manufacturer’s protocol (Millipore, USA). β-actin or GAPDH was used as a loading control. The details of all primary and second antibodies are shown in **Supplemental Table 3**.

**Quantitative reverse transcriptase polymerase chain reaction (qRT-PCR)**

Total RNA was obtained from liver biopsies of BA and controls, liver tissues of RRV and control mice, or mouse single cell derived organoids using the RNeasy Mini Kit, following the manufacturer’s protocol (Qiagen, Germany). For harvesting mouse single cell derived organoids from 4-well plates, 1 mL of ice-cold D-PBS was first added to each well, and the mixture of Matrigel and organoids was transferred to a 15 mL centrifuge tube. Subsequently, a minimum of 10 mL of ice-cold wash buffer (advanced DMEM/F12 supplemented with 1% penicillin-streptomycin) was added to the tube, followed by incubation on ice for 10 min and centrifugation at 300 ×g for 5 min at 4℃. The pellet was then resuspended in 10 mL of ice-cold wash buffer and centrifuged again under the same conditions (300 ×g, 5 min, 4℃) to thoroughly remove residual Matrigel. This step is critical for obtaining a high yield of total RNA. For mRNA detection, total RNA (500 ng) was reverse-transcribed using a reverse transcription kit (Takara, Tokyo, Japan). qRT-PCR was performed using the ABI Prism 7900HT (Applied Biosystems, California, USA). The primer sequences used for qRT-PCR analysis were in **Supplemental Table 7**. Relative expression level was calculated by the 2^-ΔΔCt^ method. ACTB or Actb mRNA was used as an internal control.

**Flow cytometry**

PFF-treated and -untreated CPs were collected and dissociated into single-cell suspensions. CP cells were stained with anti-SOX9 and -CK19 antibodies (detailed in Supplemental Table 3) following the Foxp3 Transcription Factor Staining Buffer Set protocol (00-5523-00; Invitrogen, USA). Briefly, 1 mL of Foxp3 Fixation/Permeabilization working solution was added to each tube and incubated for 30 min at 4°C. Then, 2 mL of 1X Permeabilization Buffer was added and the samples were centrifuged at 400–600 g for 5 min at room temperature. The supernatant was discarded and the recommended amount of directly conjugated antibodies were added to resuspend the CP cells and incubated for 30 min at room temperature. After washing with 2 mL of 1X Permeabilization Buffer and centrifugation (500 g, 3 min), cells were resuspended in 500 µL of 2% PFA/PBS and analyzed using a flow cytometer (Beckman Coulter, USA).

**Reference:**

1.	Fleming SJ, Chaffin MD, Arduini A, et al. Unsupervised removal of systematic background noise from droplet-based single-cell experiments using CellBender. *Nat Methods* 2023; **20**(9): 1323-35.

2.	Stuart T, Butler A, Hoffman P, et al. Comprehensive Integration of Single-Cell Data. *Cell* 2019; **177**(7): 1888-902 e21.

3.	Xu C, Lopez R, Mehlman E, Regier J, Jordan MI, Yosef N. Probabilistic harmonization and annotation of single-cell transcriptomics data with deep generative models. *Mol Syst Biol* 2021; **17**(1): e9620.

4.	Song Y, Miao Z, Brazma A, Papatheodorou I. Benchmarking strategies for cross-species integration of single-cell RNA sequencing data. *Nat Commun* 2023; **14**(1): 6495.

5.	Gulati GS, Sikandar SS, Wesche DJ, et al. Single-cell transcriptional diversity is a hallmark of developmental potential. *Science* 2020; **367**(6476): 405-11.

6.	Li B, Dewey CN. RSEM: accurate transcript quantification from RNA-Seq data with or without a reference genome. *BMC Bioinformatics* 2011; **12**: 323.

7.	Badia IMP, Velez Santiago J, Braunger J, et al. decoupleR: ensemble of computational methods to infer biological activities from omics data. *Bioinform Adv* 2022; **2**(1): vbac016.

8.	Zhu Z, Lu C, Lv X, et al. EP300/YAP1-SERPINE1 Signaling Regulates Ductular Reaction and Liver Fibrosis in Biliary Atresia. *Cell Mol Gastroenterol Hepatol* 2026; **20**(1): 101640.
