# Supplementary material for: Expanded Hepatic Progenitor Cells Featured with Aggregation of α‐Synuclein Contribute to Pathologic Bile Duct Regeneration in Biliary Atresia
Source: Adv Sci (Weinh). 2026 Jun 29:e76054. Online ahead of print. doi: 10.1002/advs.76054 (PMC13336565; doi:10.1002/advs.76054)
Supplement: Supplementary file 2 — Supporting File 2: advs76054‐sup‐0002‐SuppMat.docx. [file ADVS-9999-e76054-s002.docx]

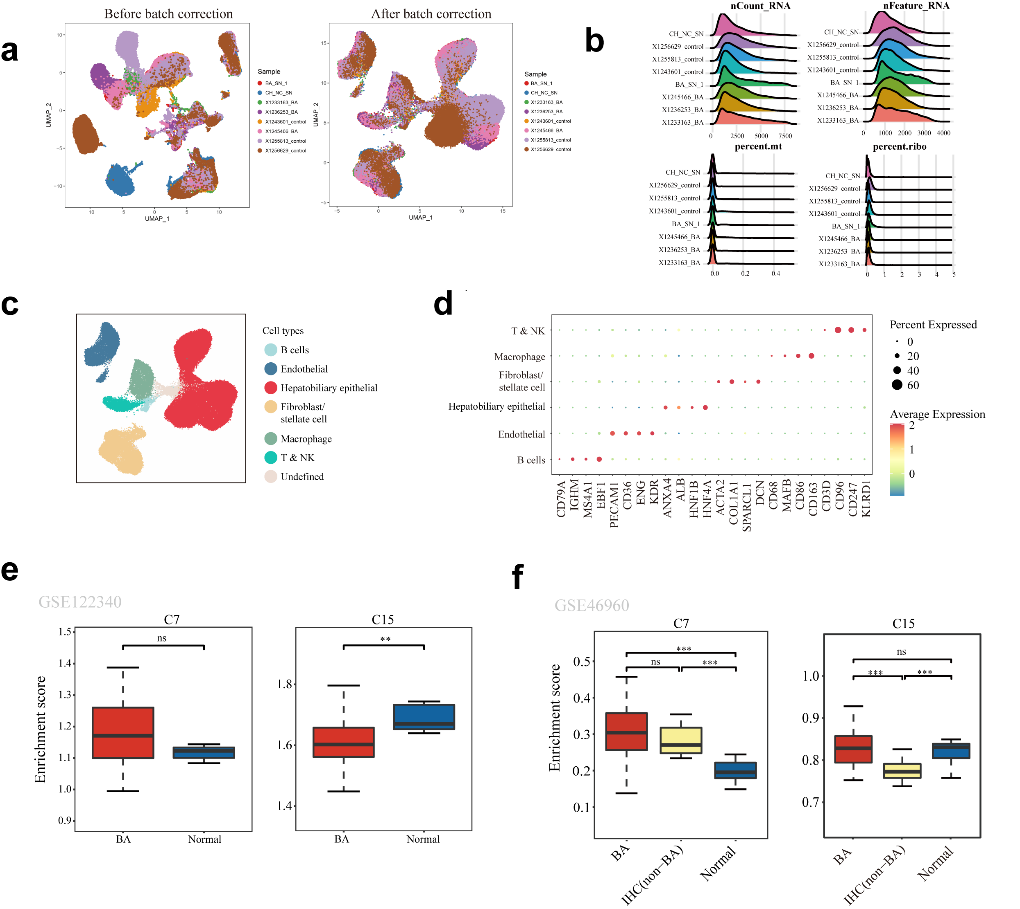


**Figure S1：snRNA-seq of BA and control liver samples.**

**(a)** All cells without correction showed significant batch effect. After correction using BBKNN, batch effects were eliminated. **(b)** Standard Seurat/Scanpy quality control plots. BA_SN_1, X1233163_BA, X1236253_BA, and X1245466_BA represent 4 BA samples respectively; X1255813_control, X1256629_control, CH_NC_SN, and X1243601_control represent 4 CC samples respectively. **(c)** UMAP visualization of all cell types from integrated BA and control samples colored according to cluster. **(d)** Bubble plot showing the expression of marker genes for the identification of different cell types for the annotation of clusters. **(e)** Analysis of DEGs of cluster 7 (C7) and cluster 15 (C15) in the extended dataset GSE122340 (171 BA and 7 normal liver tissues). *P* value was calculated by wilcoxon rank-sum test. **(f)** Analysis of DEGs of cluster 7 (C7) and cluster 15 (C15) in the extended dataset GSE46960 (64 cases of BA, 14 cases of non-BA intrahepatic cholestatic liver tissues, and 7 cases of normal liver tissues). *P* value was calculated by wilcoxon rank-sum test. **p* < 0.05; ***p* < 0.01; ****p* < 0.001, ns: not significant.


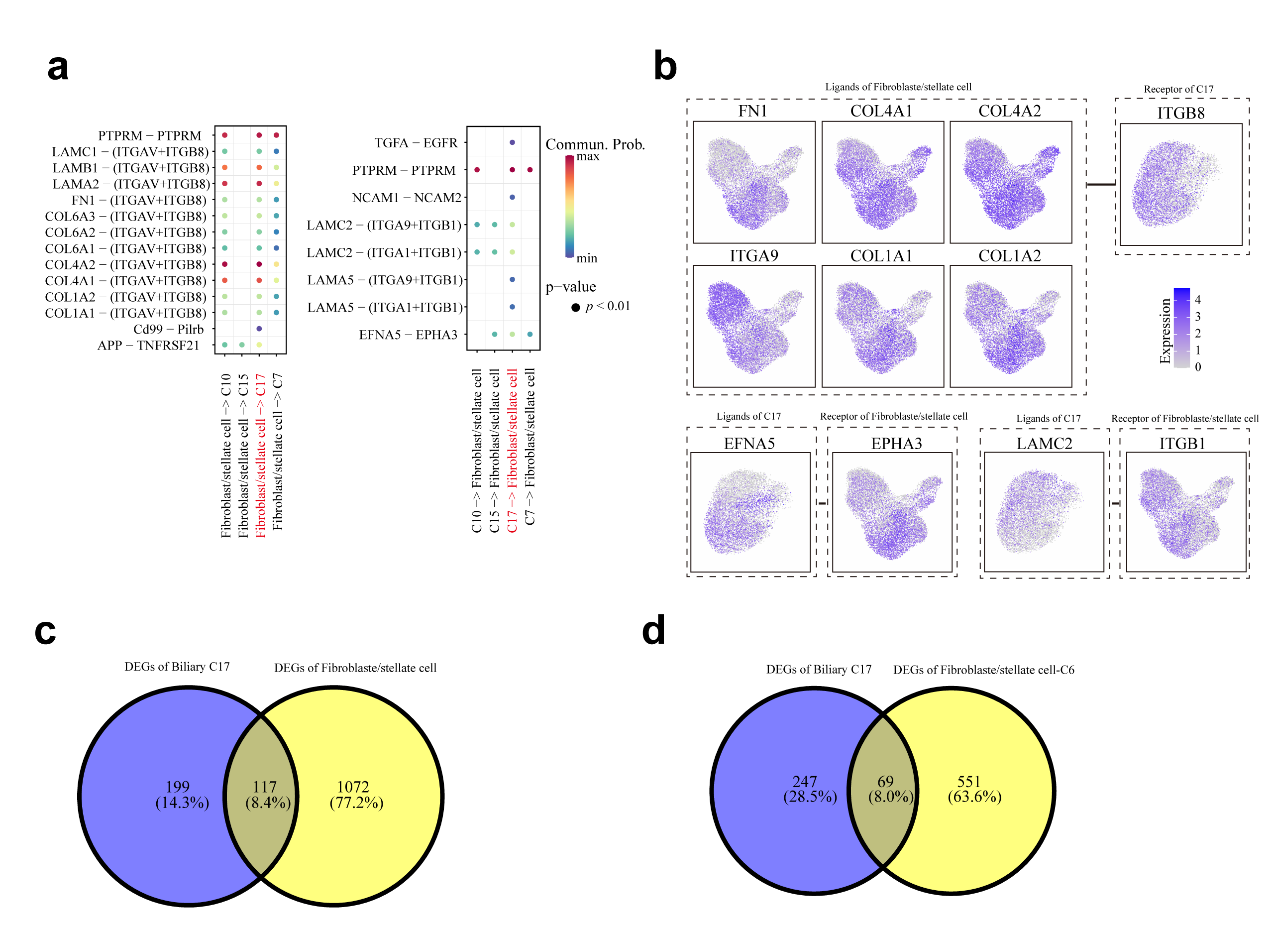


**Figure S2:** **Ligand-receptor interactions and DEGs between biliary clusters and** **fibroblast/stellate cells.**

**(a)** Pairs of ligand-receptors between biliary clusters and fibroblast/stellate cells. The ligands were mainly family members of laminins and collagens, while the receptors were mainly from integrins. **(b)** Feature plot of major ligands and receptors in fibroblast/stellate cells or biliary cluster 17 (C17). Venn diagram of overlapped genes between **(c)** DEGs (log_2_FC>0.25 and adjusted *p*<0.05) in C17 and the signature of fibroblast/stellate cells (DEGs with log_2_FC>0.25 and adjusted *p*<0.05) and **(d)** DEGs (log_2_FC>0.25 and adjusted *p*<0.05) in C17 and the signature of fibroblast/stellate cells cluster 6 (C6) (DEGs with log_2_FC>0.25 and adjusted *p*<0.05).


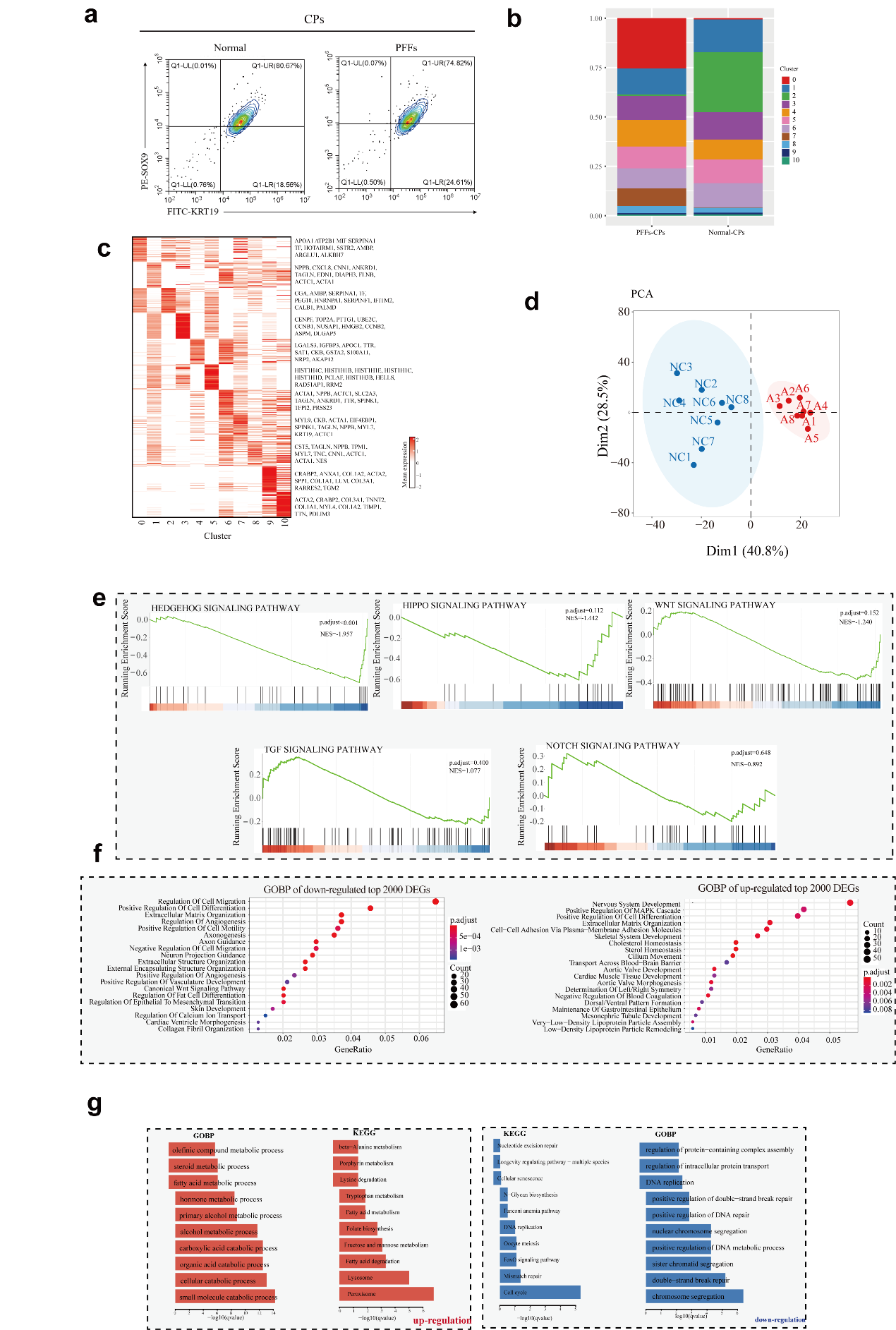


**Figure S3: iPSC derived CLC organoids with or without PFFs.**

**(a)** Flow cytometric analysis for KRT19 and SOX9 in PFFs-treated (PFFs) and untreated (Normal) CPs. **(b)** The proportion of each cluster in PFFs-treated (PFFs-CPs) or untreated normal CPs (Normal CPs). **(c)** Heatmap showing top10 DEGs (log_2_FC>0.25 and adjusted *p*<0.05) of each cluster from scRNA-seq at CPs stage. **(d)** PCA plot showing clustering of transcriptomes of PFFs-treated (A1 to A8) and untreated (NC1-NC8) CLC organoids (n of each group=8). **(e)** Gene Set Variation Analysis of pathways (Hippo, TGF, WNT, Hedgehog, and Notch) in DEGs between PFFs-treated and untreated CLC organoids. **(f)** Gene Ontology analysis on the top 2000 up-regulated or down-regulated DEGs (adjusted *p*<0.05) of CLC organoids from smart-seq data. **(g)** Gene Ontology analysis and KEGG pathway enrichment analysis on the up-regulated or down-regulated DEGs (adjusted *p*<0.05) of BA liver tissue derived organoids from smart-seq data.
