# Supplementary material for: Expanded Hepatic Progenitor Cells Featured with Aggregation of α‐Synuclein Contribute to Pathologic Bile Duct Regeneration in Biliary Atresia
Source: Adv Sci (Weinh). 2026 Jun 29:e76054. Online ahead of print. doi: 10.1002/advs.76054 (PMC13336565; doi:10.1002/advs.76054)
Supplement: Supplementary file 3 — Supporting File 3: advs76054‐sup‐0003‐TablesS1‐S7.docx. [file ADVS-9999-e76054-s001.docx]

**Supplementary Table 1: Clinical information of liver biopsies for single-nucleus RNA sequencing.**

|  | BA | | | | CC | | | |
| --- | --- | --- | --- | --- | --- | --- | --- | --- |
|  |  |  |  |  | Ctrl1 | | Ctrl2 | |
| Sex | Female | Female | Male | Male | Female | Male | Female | Male |
| Age (Days) | 68 | 63 | 85 | 64 | 66 | 51 | 170 | 116 |
| ALT (U/L) | 72 | 481 | 162 | 95 | 117 | 50 | 27 | 20 |
| AST (U/L) | 90 | 454 | 178 | 161 | 104 | 86 | 29 | 21 |
| GGT (U/L) | 436 | 260 | 907 | 1954 | 361 | 762 | 189 | 111 |
| DBIL (µmol/L) | 173.13 | 185.3 | 195.39 | 153.82 | 87.73 | 85.08 | 22.19 | 21.5 |

BA: Biliary atresia; CC: choledochal cyst; Ctrl2: CC without severe cholestasis; Ctrl 1: CC with severe cholestasis; ALT: alanine transaminase; AST: aspartate transaminase; GGT: gamma-glutamyl transferase; DBIL: direct bilirubin.

**Supplementary Table 2: Clinical characteristic of fresh liver biopsy samples from three patients with BA for** **NCAM1^+^ EpCAM^+^ and NCAM1^-^ EpCAM^+^ cells derived organoids culture.**

| Sample ID | Sex | Age at surgery (Days) | ALT (U/L) | AST (U/L) | GGT (U/L) | DBIL (µmol/L) |
| --- | --- | --- | --- | --- | --- | --- |
| BA1 | Female | 60 | 154 | 165 | 240 | 135.87 |
| BA2 | Male | 85 | 89 | 144 | 492 | 83.01 |
| BA3 | Female | 44 | 35 | 49 | 432 | 46.59 |

BA: Biliary atresia; ALT: alanine transaminase; AST: aspartate transaminase; GGT: gamma-glutamyl transferase; DBIL: direct bilirubin.

**Supplementary Table 3. List of primary and secondary antibodies**

| Antibody | Host species | Manufacturer | Catalog # | Dilution | RRID |
| --- | --- | --- | --- | --- | --- |
| KRT19（CK19） | Mouse | Bioss | bsm-33057M | IF: 1:250 | AB_3716580 |
| α-synuclein | Rabbit | Abcam | Ab209538 | IF: 1:5000 | AB_2714215 |
| α-synuclein | Rabbit | Abcam | Ab212184 | WB: 1:1000 | AB_2941889 |
| NCAM1 | Rabbit | Abcam | ab313779 | IF:1:500  IHC:1:2000 | AB_3095734 |
| EpCAM | Rabbit | Abcam | Ab223582 | IF: 1:1000 | AB_2762366 |
| β-actin | Rabbit | Abcam | Ab8227 | WB: 1:1000 | AB_2305186 |
| GAPDH | Mouse | Proteintech | 60004-1-Ig | WB: 1:1000 | AB_2107436 |
| Alexa Fluor® 488 conjugated Cytokeratin 19 | Rabbit | Abcam | Ab192643 | FC:1/500 | AB_2927708 |
| PE conjugated SOX9 | Rabbit | Abcam | Ab224019 | FC:1:1000 | AB_3716581 |
| YSFluor™ 594 Goat Anti-Mouse IgG(H+L) |  | YEASEN | 33212ES60 | 1:200 | AB_3096072 |
| YSFluor™ 488 Goat Anti-Mouse IgG(H+L) |  | YEASEN | 33206ES60 | 1:200 | AB_3662603 |
| YSFluor™ 594 Goat Anti-Rabbit IgG(H+L) |  | YEASEN | 33112ES60 | 1:200 | AB_3661961 |
| YSFluor™ 488 Goat Anti-Rabbit IgG(H+L)  Goat anti-Rabbit IgG (H+L) Cross-Adsorbed Secondary Antibody, Alexa Fluor™ 594  Goat anti-Mouse IgG (H+L) Cross-Adsorbed Secondary Antibody, Alexa Fluor™ 488 |  | YEASEN  Invitrogen  Invitrogen | 33106ES60  A-11012  A-11001 | 1:200  1:1000  1:1000 | AB_3697920  AB_141359  AB_2534069 |

**Supplementary Table 4: Clinical characteristic of the samples for serum total α-synuclein detection.**

|  | **BA(n=40)** | **Control(n=20)** | ***P**** |
| --- | --- | --- | --- |
| Age (days) | 58.50 (44.50-69.75) | 98.50 (34.25-172.50) | 0.010 |
| Sex (n) |  |  | 0.264 |
| Male | 18 | 6 |  |
| Female | 22 | 14 |  |
| Serum total α-synuclein (ng/mL) | 2.456±0.949 | 1.368±1.061 | <0.001 |
| DBIL (µmol/L) | 114.40(90.46-114.60) | 2.86(2.32-5.47) | <0.001 |
| GGT (U/L) | 425.00 (227.80-758.30) | 43.00 (26.25-112.00) | <0.001 |

*P value: Sex was tested using χ2 test. Serum total α-synuclein was compared by student t test. All other rows were tested using Mann‐Whitney U test. GGT: gamma-glutamyl transferase; DBIL: direct bilirubin. Control samples contain 18 patients with choledochal cyst (CC) and 2 patients with neonatal intrahepatic cholestasis (IHC).

**Supplementary Table 5. List of reagents for iPSC maintenance and cholangiocyte-like cell organoid differentiation.**

| Reagents | Manufacturer | Catalog # |
| --- | --- | --- |
| Poly(Vinyl Alcohol) (PVA) 87–90% Hydrolyzed | Sigma-Aldrich | P8136-250G |
| IMDM | Life technologies | 12440046 |
| Gelatin | Sigma-Aldrich | G9391-100G |
| Water for embryo transfer | Sigma-Aldrich | W1503 |
| Ham’s F-12 Nutrient Mix, GlutaMAX Supplement | Life technologies | 31765068 |
| Concentrated lipids | Life technologies | 11905031 |
| Monothioglycerol | Sigma-Aldrich | M6145 |
| Insulin | Sigma-Aldrich | I9278 |
| Transferrin | Sigma-Aldrich | T8158 |
| P/S | Life technologies | 15140122 |
| L-Glutamine | Life technologies | A2916801 |
| β-Mercaptoethanol | Sigma-Aldrich | M6250-10ml |
| Advanced DMEM/F12 | Life technologies | 12634010 |
| FBS | Life technologies | A5669701 |
| Collagenase IV | Life technologies | 17104019 |
| Dispase | Life technologies | 17105041 |
| DMEM/F12 | Life technologies | 11330032 |
| KOSR | Life technologies | 10828028-500ml |
| Recombinant human activin A | RD system | 338-AC-050 |
| Recombinant human FGF basic | RD system | 233-FB-025 |
| Recombinant human BMP-4 | RD system | 314-BP-020 |
| LY 294002 | RD system | 1130/25 |
| CHIR99021 | RD system | 4423/50 |
| [SB 431542](https://www.rndsystems.com/cn/products/sb-431542_1614) | RD system | 1614/10 |
| Recombinant Human Keratinocyte Growth Factor-2（FGF10） | RD system | 345-FG-250 |
| Retinoic acid | Sigma-Aldrich | R2625-500mg |
| MatrigeL | Corning | 356237 |
| MatrigeL | Corning | 356231 |
| MEM nonessential amino acids | Gibco | 11140050-100ml |
| RPMI 1640 | Gibco | 61870036 |
| B-27™ (50X) | Gibco | 17504044 |
| Y-27632 (ROCK inhibitor) | Selleck | S1049-10mg |
| Cell dissociation buffer, enzyme-free, PBS | Gibco | 13151014 |

**Supplementary Table 6. List of** **reagents for organoid culture medium.**

| Reagents | Catalogue number | Final concentration/volume |
| --- | --- | --- |
| Advanced DMEM/F12 | Gibco #12634-010 | 9.4 mL |
| Nicotinamide | Sigma#N0636 | 10 mM |
| Glutamax | Gibco #35050-061 | 100μL |
| N-acetylcysteine | MCE#HY-B0215 | 1.25 mM |
| HEPES | Beyotime#C0215 | 250μL |
| B27 | Gibco #17504-044 | 200μL |
| N2 | Gibco #17502-048 | 100μL |
| R-spondin | R&D#7150-RS-50 | 500 ng/mL |
| Wnt-3a | Peprotech#315-20 | 100 ng/mL |
| mEGF | Invitrogen#PMG8045 | 50 ng/mL |
| FGF10 | Peprotech#100-26 | 100 ng/mL |
| HGF | R&D#294-HG-005 | 50 ng/mL |
| mNoggin | R&D#1967-NG-100 | 100 ng/mL |
| Gastrin | MCE#HY-P2671 | 10 nM |
| A83-01 | Selleck#S7692 | 5 μM |
| Foskolin | Selleck#S2449 | 10 μM |
| Antibiotic-Antimycotic | Gibco#15240-062 | 100μL |
| ROCK- inhibitor | Sigma #SCM075 | 10 μM |

**Supplementary Table 7. Sequences of primers for RT-PCR.**

| **Gene symbol** | | |
| --- | --- | --- |
| **NCAM1** | Forward | 5’-GGCATTTACAAGTGTGTGGTTAC-3’ |
| **SNCA** | Reverse  Forward  Reverse | 5’- TTGGCGCATTCTTGAACATGA -3’  5’-AAGAGGGTGTTCTCTATGTAGGC-3’  5’- GCTCCTCCAACATTTGTCACTT -3’ |
| **Ncam1** | Forward | 5’-ACCACCGTCACCACTAACTCT -3’ |
|  | Reverse | 5’-TGGGGCAATACTGGAGGTCA -3’ |
| **Snca** | Forward | 5’-GCAAGGGTGAGGAGGGGTA -3’ |
|  | Reverse | 5’-CCTCTGAAGGCATTTCATAAGCC-3’ |
| **Epcam** | Forward | 5’-GCGGCTCAGAGAGACTGTG-3’ |
|  | Reverse | 5’-CCAAGCATTTAGACGCCAGTTT-3’ |
| **Krt19** | Forward | 5’-GGGGGTTCAGTACGCATTGG -3’ |
|  | Reverse | 5’-GAGGACGAGGTCACGAAGC -3’ |
| **Hnf1b** | Forward | 5’-AGGGAGGTGGTCGATGTCA -3’ |
|  | Reverse | 5’-TCTGGACTGTCTGGTTGAACT -3’ |
| **Ggt1** | Forward | 5’-TTTGTCATCATCGGCCTCTGT -3’ |
|  | Reverse | 5’-CCCGTCCAATCTCTGAGCAG -3’ |
| **Sox9** | Forward | 5’-GAGCCGGATCTGAAGAGGGA -3’ |
|  | Reverse | 5’-GCTTGACGTGTGGCTTGTTC -3’ |
| **Alb** | Forward | 5’-TGCTTTTTCCAGGGGTGTGTT -3’ |
|  | Reverse | 5’-TTACTTCCTGCACTAATTTGGCA -3’ |
| **Hnf4a** | Forward | 5’-CACGCGGAGGTCAAGCTAC -3’ |
|  | Reverse | 5’-CCCAGAGATGGGAGAGGTGAT -3’ |
| **Actb** | Forward | 5’-GGCTGTATTCCCCTCCATCG-3’ |
|  | Reverse | 5’-CCAGTTGGTAACAATGCCATGT-3’ |
| **ACTB** | Forward | 5’- CATGTACGTTGCTATCCAGGC-3’ |
|  | Reverse | 5’- CTCCTTAATGTCACGCACGAT-3’ |
