# Supplementary material for: Expanded Hepatic Progenitor Cells Featured with Aggregation of α‐Synuclein Contribute to Pathologic Bile Duct Regeneration in Biliary Atresia
Source: Adv Sci (Weinh). 2026 Jun 29:e76054. Online ahead of print. doi: 10.1002/advs.76054 (PMC13336565; doi:10.1002/advs.76054)
Supplement: Supplementary file 5 — Supporting File 5: advs76054‐sup‐0005‐Data.pdf. [file ADVS-9999-e76054-s003.pdf]

Loiding samples： Marker-PBS treated CPs-PFFs treated CPs-Marker-PBS treated CPs-PFFs treated CPs

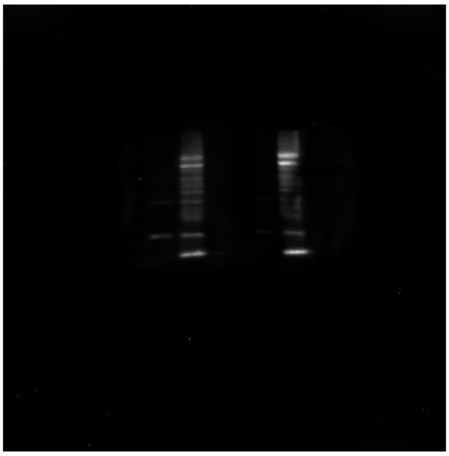

α-synuclein

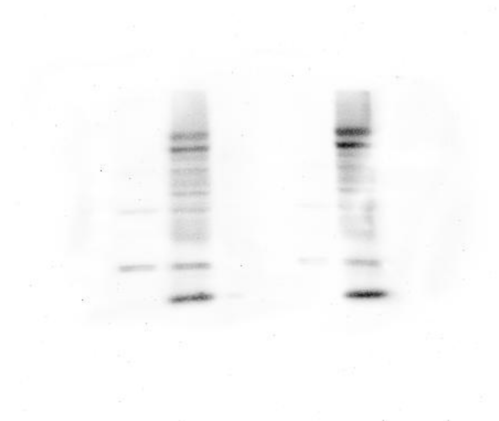

α-synuclein inverted

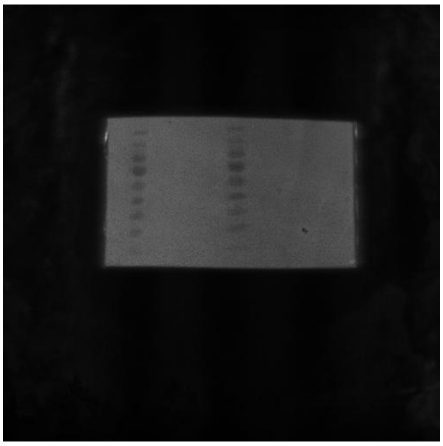

Marker

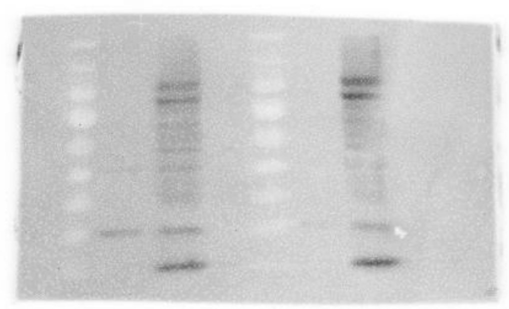

α-synuclein +Marker

Loiding samples：Marker-PBS treated CPs-PFFs treated CPs-Marker-PBS treated CPs-PFFs treated CPs

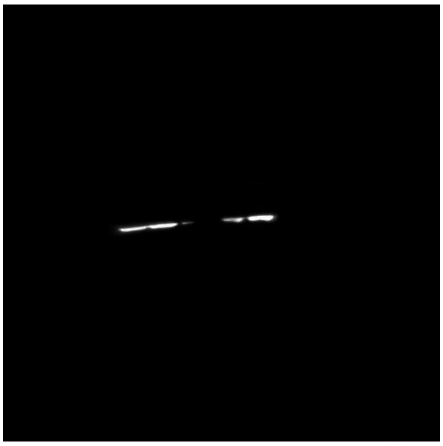

β-actin

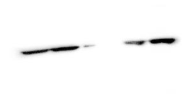

β-actin inverted

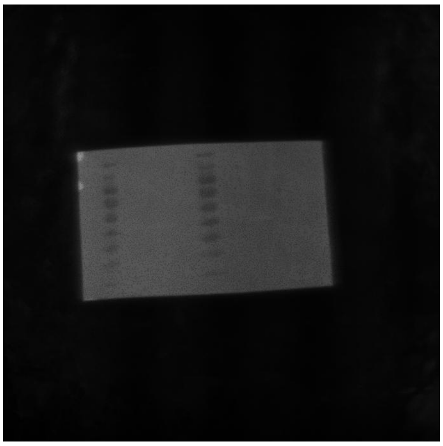

Marker

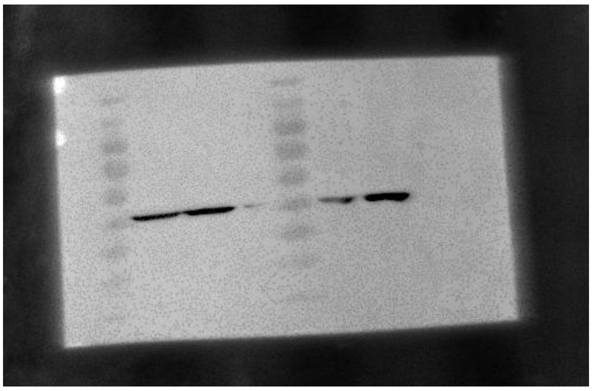

β-actin +Marker

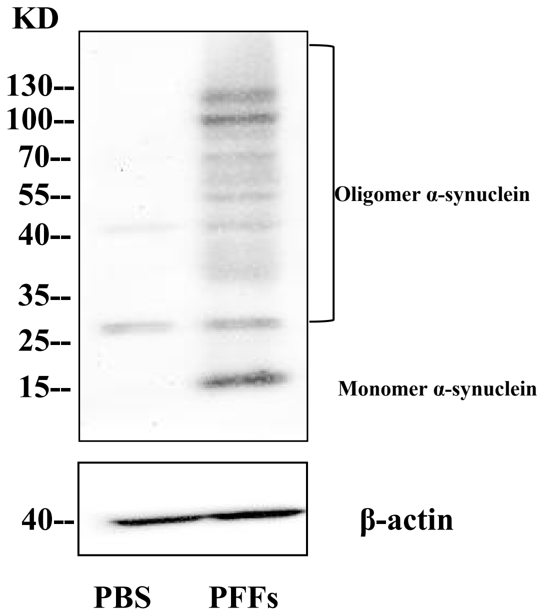

KD
130--
100--
70--
55--
40--
35--
25--
15--
Oligomer α-synuclein
Monomer α-synuclein
40--
β-actin
PBS
PFFs

Loiding samples: Marker-Ctrl-Ctrl-Ctrl-SNCA-OE-SNCA-OE-SNCA-OE-Marker

GAPDH

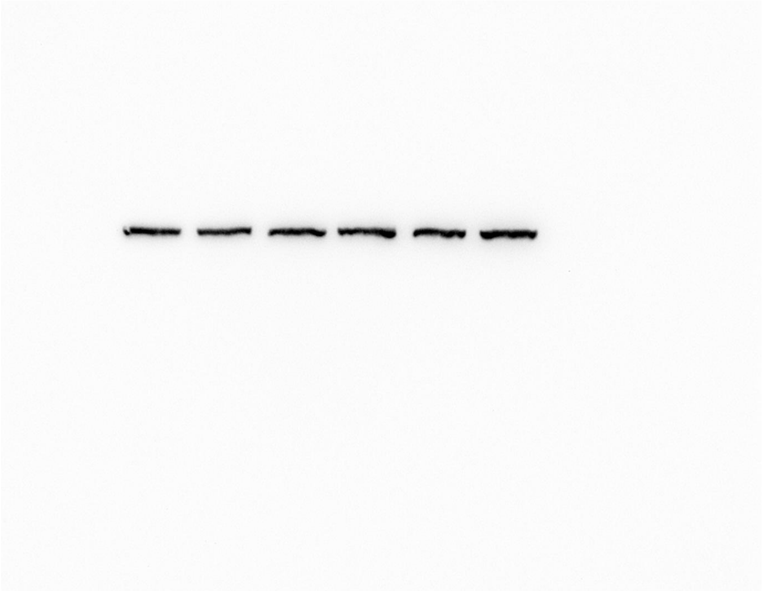

GAPDH with marker

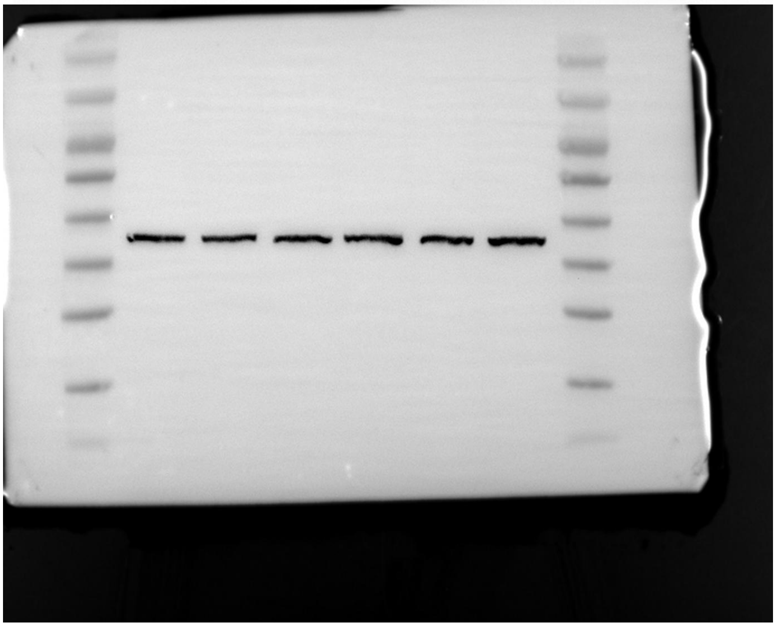

α-synuclein

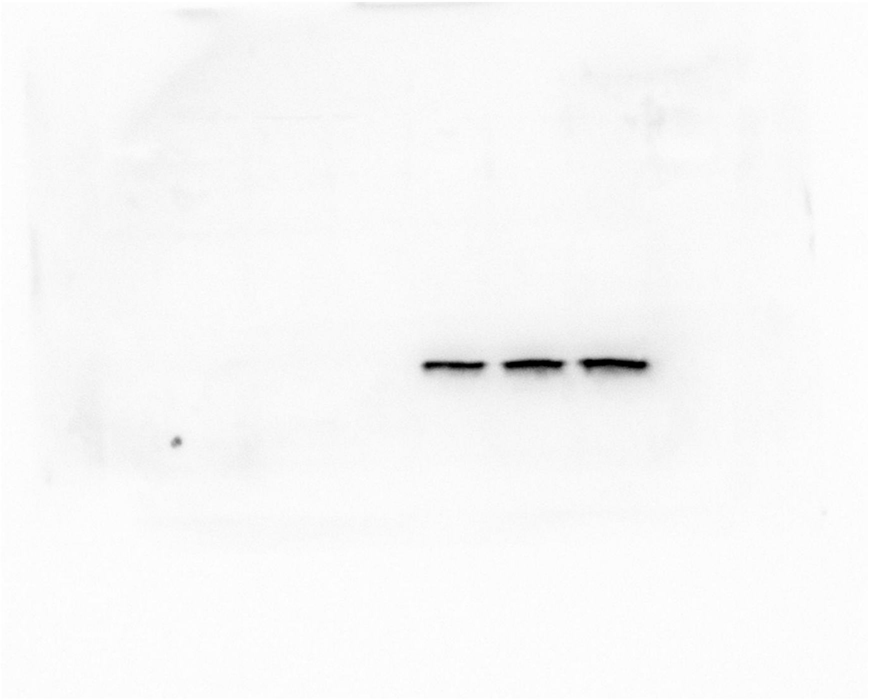

α-synuclein with marker

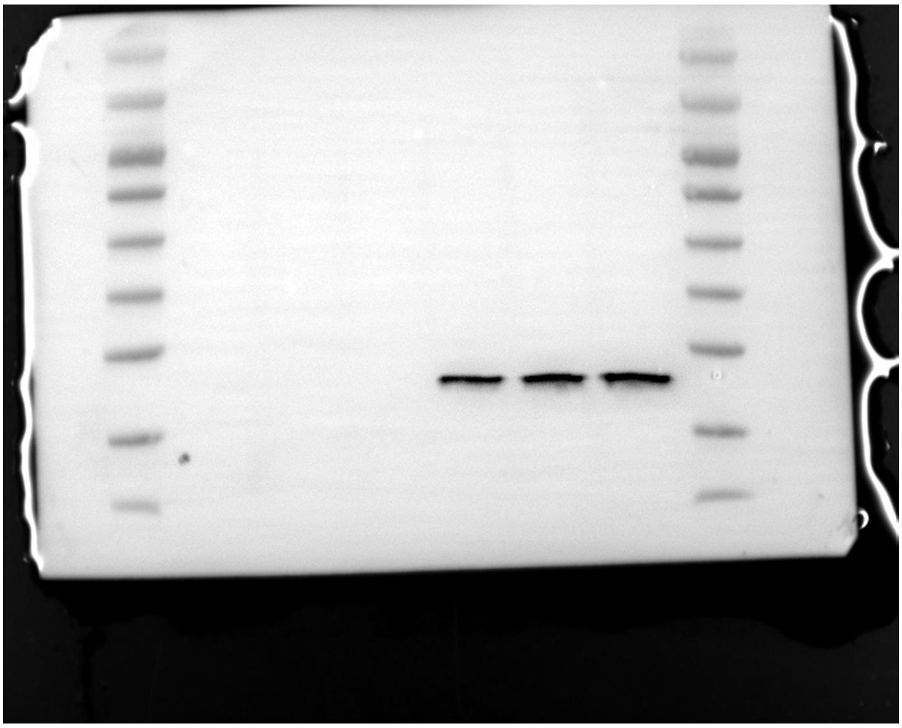

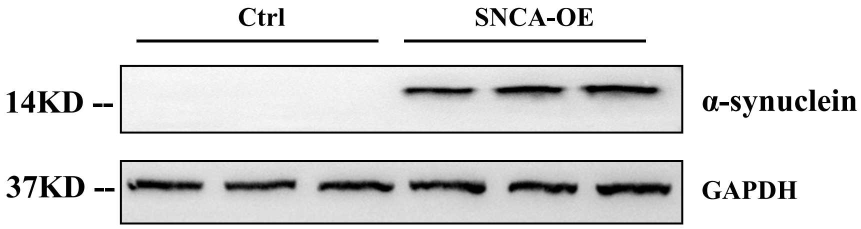
Ctrl
SNCA-OE
14KD --
α-synuclein
37KD --
GAPDH
